# Multitarget and suspect screening of antimicrobials in soil and manure by means of QuEChERS — liquid chromatography tandem mass spectrometry

**DOI:** 10.1007/s00216-023-04905-2

**Published:** 2023-08-23

**Authors:** I. Vergara-Luis, N. Bocayá, M. Irazola-Duñabeitia, O. Zuloaga, M. Lacuesta, M. Olivares, A. Prieto

**Affiliations:** 1 Department of Analytical Chemistry, Faculty of Science and Technology, University of the Basque Country (UPV/EHU), 48490 Leioa, Basque Country Spain; 2 Research Centre for Experimental Marine Biology and Biotechnology (PIE), University of the Basque Country (UPV/EHU), Plentzia, Basque Country Spain; 3 Department of Plant Biology and Ecology, Faculty of Pharmacy, University of the Basque Country (UPV/EHU), Vitoria-Gasteiz, Basque Country Spain

**Keywords:** Antimicrobials, Soil, Manure, UHPLC-MS/MS, q-Orbitrap

## Abstract

The present work aimed to develop an accurate analytical method for the simultaneous analysis of twenty-four antimicrobials in soil:compost and animal manure samples by means of ultra-high performance liquid chromatography coupled to a triple-quadrupole mass spectrometer (UHPLC-QqQ). For this purpose, the effectiveness of two extraction techniques (i.e. focused ultrasound solid–liquid extraction (FUSLE) and QuEChERS (quick, easy, cheap, effective, rugged and safe)) was evaluated, and the clean-up step using solid-phase extraction (SPE) was also thoroughly studied. The method was successfully validated at 10 μg·kg^−1^, 25 μg·kg^−1^, and 50 μg·kg^−1^ showing adequate trueness (70–130%) and repeatability (RSD < 30%), with few exceptions. Procedural limits of quantification (LOQ_PRO_) were determined for soil:compost (0.45 to 7.50 μg·kg^−1^) and manure (0.31 to 5.53 μg·kg^−1^) samples. Pefloxacin could not be validated at the lowest level since LOQ_PRO_ ≥ 10 μg·kg^−1^. Sulfamethazine (7.9 ± 0.8 µg·kg^−1^), danofloxacin (27.1 ± 1.4 µg·kg^−1^) and trimethoprim (4.9 ± 0.5 µg·kg^−1^) were detected in soil samples; and tetracycline (56.8 ± 2.8 µg·kg^−1^), among other antimicrobials, in the plants grown on the surface of the studied soil samples. Similarly, sulfonamides (SAs), tetracyclines (TCs) and fluoroquinolones (FQs) were detected in sheep manure in a range of 1.7 ± 0.3 to 93.3 ± 6.8 µg·kg^−1^. Soil and manure samples were also analysed through UHPLC coupled to a high-resolution mass-spectrometer (UHPLC-qOrbitrap) in order to extend the multitarget method to suspect screening of more than 22,281 suspects. A specific transformation product (TP) of sulfamethazine (formyl-sulfamethazine) was annotated at 2a level in manure samples, among others. This work contributes to the efforts that have been made in the last decade to develop analytical methods that allow multitarget analysis of a wide variety of antimicrobials, including TPs, which is a complex task due to the diverse physicochemical properties of the antimicrobials.

**Supplementary information:**

The online version contains supplementary material available at 10.1007/s00216-023-04905-2.

## Introduction

The growing human population has generated the need of developing different sustainable agriculture strategies to cover the increasing food demands [1]. In that sense, trying to reduce the use of mineral fertilisers in crop production enhancement, the use of organic fertilisers, such as animal manure and manure-based compost, has increased [2, 3]. Manure is regarded as a very valuable fertiliser as it contains essential nutrients for plant growth [4]. However, the massive use of pharmaceuticals, especially antimicrobials, to fight against bacterial infections in intensive livestock farming has led to consider animal manure and derived compost as important pathways for the entry of antimicrobials into the soil environment and their consequent accumulation in plants [4–7]. Diverse scientific works have reported the occurrence of residual antimicrobials in animal wastes worldwide as consequence of incomplete metabolisation and partial absorption of these drugs [8–13].

The scarce legislation about the occurrence of antimicrobials in manure and soils enlarges the problem. The closest detail to regulation is a note for guidance provided by the European Agency for the Evaluation of Medicinal Products in which it is stated that the content of drug residues should not exceed 100 µg·kg^−1^ in manure destined to land application and 10 µg·kg^−1^ in the soil fertilised with manure [12–14].

Nevertheless, the major problem associated to the presence of pharmaceuticals in the environment is the spread of antimicrobial resistance [5, 9] since it could cause the ineffectiveness of currently available antimicrobials against common infections [6] posing a potential threat to human health. Even if antimicrobials are detected at low concentration levels (i.e. µg·kg^−1^ levels) in manure and soils [14], low concentrations have shown to be enough to cause genetic changes in bacterial genomes so that bacteria in soils may represent a reservoir of resistance genes that could be transferred to human pathogens [10, 15]. Moreover, antimicrobials tend to degrade under different light, humidity, and temperature conditions [10, 16] resulting in the formation of a variety of transformation products (TPs), some of which have shown greater toxicity than the parent compound [17]. So far, little is known about whether TPs are involved in the generation of resistance; therefore, in order to perform a comprehensive risk assessment, it is of utmost importance to develop analytical methodologies to detect trace antimicrobials and monitor TPs formation in manure, compost (before it is considered suitable for soil application) and soils.

Although there are several analytical methods intended to organic contaminants analysis in biosolids, those involving antimicrobial analysis have only been developed in recent years. In those methods, liquid chromatography tandem mass spectrometry (UHPLC-MS/MS) is the most used analytical technique for antimicrobials’ quantification [18, 19], but different sample treatments are used. Commonly, antimicrobials are extracted by ultrasound-assisted solvent extraction (USE) [20], focused ultrasound solid–liquid extraction (FUSLE), pressurized liquid extraction (PLE) [21, 22] or microwave-assisted extraction (MAE), among others [18]. Recently, QuEChERS [23] (quick, easy, cheap, effective, rugged and safe), a miniaturised extraction and clean-up technique, is gaining interest due to it allows performing high-throughput analyses, demonstrating advantages in terms of time and laboratory resources savings. Regardless the extraction method used, the extract is often submitted to a clean-up step that enables the removal or minimisation of possible matrix interferences. The clean-up step is commonly performed by solid-phase extraction (SPE) using sorbents like Oasis hydrophilic-lipophilic balance (HLB) or/and strong anion exchange (SAX) [11, 20, 24, 25] cartridges.

However, the different physicochemical properties of antimicrobials difficult their simultaneous analysis in one run, being necessary the use of specific methods for the analysis of each group. In recent years, some works have focused on the development of methods that allow the simultaneous analysis of these compounds in soil and manure. Some of those methods included selected antimicrobials of the same family or few congeners of a limited number of families. For instance, the method consisted on PLE extraction followed by SPE clean-up reported by Barron et al. [26] was only validated for the analysis of three antimicrobials in soils. Similarly, another QuEChERS-based method developed by Meng et al. [27] only covers the analysis of sulfonamides (SAs) and macrolides (MCs). Other methods included a large group of antimicrobials; however, in some of those cases, the accuracy requirements of the regulations were not fully met. That is the case of the QuEChERS-based method developed by Martínez-Piernas et al. [23], which regarding antimicrobials only accomplished the accuracy and precision requirements for three MCs and trimethoprim; or in the work done by da Silva et al., which among the extensive list of studied antimicrobials, it showed some accuracy issues especially for fluoroquinolones (FQs) and tetracyclines (TCs) [28].

Within this context, the present work aimed to develop an accurate analytical method for the simultaneous analysis of five SAs, four TCs, six FQs, four MCs, one diaminopyridine (DP), mycophenolic acid (compound isolated from *Penicillium stoloniferum*), and three antifungal compounds (AFs) in animal manure and soil:compost samples by means of UHPLC-MS/MS. As noted above, non-human usage of antimicrobials affected the occurrence of resistant bacteria and thereby human exposure to them. The consequences of that exposure have been reported to be particularly severe when pathogens were resistant to antimicrobials critically important for human health. Therefore, in this work antimicrobials classified by the World Health Organization (WHO) as critically or highly important for human medicine have been studied [29]. FUSLE vs QuEChERS-based salting-out extraction techniques were compared, and the clean-up step using SPE was thoroughly evaluated in order to get the best conditions in terms of sensitivity and accuracy. Moreover, the method was extended to the determination of more antimicrobials and possible TPs by suspect analysis using high-resolution mass spectrometry. The methodologies were applied to the analysis of twenty-five soils, twenty-four plants, seven sheep manures, and nine horse manure samples acquired from different cheese producers and local farms, respectively.

## Experimental procedure

### Reagents and materials

Distributor and specific physicochemical properties for the target antimicrobials and surrogate standards are gathered in Table S1. Individual solutions were monthly prepared at 1000–3000 mg·kg^−1^ in UHPLC-quality methanol (MeOH, 99.9%, Scharlau, Sentmenat, Catalonia, Spain), UHPLC-quality acetonitrile (ACN, 99.9%, Avantor Performance Materials, Gliwice, Silesia, Poland), or dimethyl sulfoxide (DMSO, Panreac AppliChem, Darmstadt, Germany) (see Table S1). In the case of FQs, standards were individually dissolved in the corresponding solvent, and three drops of a NaOH 2 M solution (99%, Merck, Darmstadt, Hesse, Germany) were added in order to prepare the concentrated stock solution [30, 31]. Intermediate 100 mg·kg^−1^, 5 mg·kg^−1^ and 1 mg·kg^−1^ mix-solutions were prepared in ACN weekly. The most concentrated solutions were stored at -20 °C, while 5 mg·kg^−1^ and 1 mg·kg^−1^ mix were kept at 4 °C. All working standard solutions were prepared and stored in silanised amber vials to avoid photodegradation [32]. NaCl (100%) acquired from PanReac AppliChem (Castellar del Vallés, Catalonia, Spain), anhydrous citric acid H_3_Cit (99.5%) and anhydrous Na_2_HPO_4_ (98%) obtained from Scharlau and anhydrous Na_2_SO_4_ (99%) from Merck were used as extraction salts. UHPLC-grade MeOH and ACN and a citrate buffer consisting of an aqueous solution of anhydrous NaH_2_Cit (99%) and Na_2_HCit·1.5H_2_O (99%) (Honeywell Fluka, Charlotte, North Carolina, USA) were used as extractants. A phosphate buffer was also prepared using Na_2_HPO_4_ (98%, Scharlau) and NaH_2_PO_4_ from PROBUS (100%, C. Busquets, Badalona, Spain) for further dilution and pH adjustment of the extract before the clean-up. Concerning the clean-up, Primary Secondary Amine (PSA), Bondesil-C_18_ (40 μm, Agilent Technologies, Santa Clara, CA, EEUU) and Graphitized Carbon Black (GCB) (37–125 μm, Superclean ENVI-Carb, Merck) sorbents and Oasis HLB cartridges (200 and 500 mg, 6 cc, 30 μm) purchased from Waters (Milford, Massachusetts, USA) were employed. Oxalic acid (100%, Merck) was used in the final extract reconstitution.

A Multi Reax shaker by Heidolph (Schwabach, Bavaria, Germany) and a 5840R centrifuge by Eppendorf (San Sebastián de Los Reyes, Madrid, Spain) were used for sample treatment procedure.

### Soil, manure and plant samples

Twenty-five soil samples were gathered close to four different cheese producers belonging to the Idiazabal Cheese P.D.O. in Nafarroa, Araba, Bizkaia and Gipuzkoa (Basque Country). The soils sampled are pastures on which animal manure was applied and on which sheep are grazing. The number of animals varies from producer to producer, but can range from 400 to 600 sheep on average. Six samples per location (dimensions 30 × 30 × 30 cm) were collected randomly. The different plant species present on the soil surface (from each collected soil fraction) were also compiled. Another soil sample was acquired from Neiker Research Centre (Biscay, Basque Country, Spain)**.** Samples were stored in a zip bag and frozen at − 80 °C when arrived at the laboratory.

As regards to manure samples, sheep manure was compiled from four different cheese producers belonging to the Idiazabal Cheese P.D.O. where the soil samples were taken. At each site, two samples were collected during two consecutive weeks, with the exception of Nafarroa producers, where only one sample was collected. Nine horse manure samples were also gathered from a local farm supplier. All manure samples were stored in a zip bag and frozen at − 80 °C until analysis.

### Sample pre-treatment and extraction

Two different extraction strategies were studied and compared in this work: QuEChERS and FUSLE. Both techniques were firstly evaluated using an antimicrobial free soil:compost (97.25:2.75, w:w) sample, and the optimum protocol was later employed for the determination of antimicrobials in manure too.

Plant samples were extracted using a modified analytical method, based on QuEChERS extraction, previously developed by the research group (see supplementary material) [33].

#### QuEChERS

QuEChERS protocol was optimised following the initial conditions established in a previous work by the research group [33]. Briefly, 5 g of fresh and homogenised soil:compost mixture, spiked with 50 µL of a 5 mg·kg^−1^ stock solution (maintained at least 30 min in darkness before extraction), were extracted with 2 mL Milli-Q water, 10 mL of ACN, 4 g of anhydrous Na_2_SO_4_ (instead of MgSO_4_, as it liberates energy that might affect the stability of the tested drugs) [34] and 1 g NaCl. The extraction pH was assessed according to what it has been observed in the literature [35]. Concretely, the extraction efficiency was tested at different pHs by the addition of (i) 0.5 g of anhydrous H_3_Cit, (ii) 0.1 g of sodium acetate or (iii) 1.25 g of sodium acetate which were mixed with 0.05 g anhydrous Na_2_HPO_4_ to get a pH 2.5, pH 4 and pH 7.5, respectively. A ceramic homogeniser was added to the mixture, and it was then shaken manually and degasified until no gas was released. All samples were eventually vortexed (2000 cycles·min^−1^, 8 min) and centrifuged (4000 cycles·min^−1^, 5 min) at 10–15 °C.

The extraction of antimicrobials in manure was performed using the optimal conditions get for soil:compost samples (pH 2.5). Owing to the higher amount of organic matter present in manure [18], 2 g of fresh manure were extracted using 760 µL of Milli-Q water, 5 mL of ACN, 2 g of anhydrous Na_2_SO_4_, 0.5 g NaCl, 0.25 g anhydrous H_3_Cit and 0.025 g Na_2_HPO_4_ in order to maintain the same sample/extraction reagent proportion as for soil:compost sample.

#### FUSLE

Dry soil:compost samples (0.5 g) spiked at 10 µg·kg^−1^ concentration level were analysed in order to compare FUSLE and QuEChERS extraction approaches. For the extraction, 7 mL of MeOH were added, and the extraction was performed during 2 min (0.8 s on and 0.2 s off every second of extraction) at 20% amplitude and 2 min of extraction time according to the experience of the research group [36–38]. The samples were then centrifuged for 12 min, at 10 °C and 10,000 cycles·min^−1^.

### Clean-up procedure

Regardless of the extraction procedure used, a clean-up of the extract was required prior to UHPLC-MS/MS analysis. In order to fulfil this cleaning purpose, SPE technique was used.

In the case of soil:compost matrix, several SPE factors were optimised: (i) the amount of the sorbent (200 or 500 mg) together with the loading volume of the extract (3.5 mL diluted to 70 mL with citrate buffer (0.05 mol·L^−1^, pH 4) in 200 mg cartridges or 7 mL diluted to 140 mL in 500 mg cartridges) and (ii) the pH of the extract (pH 4 or pH 7.5). Under optimal conditions, an aliquot of 3.5 mL of the extract was isolated, diluted to 70 mL and loaded to 200 mg Oasis HLB cartridges, which were previously conditioned with 5 mL of ACN, 5 mL of Milli-Q water and 5 mL of citrate buffer (pH = 4). Once the diluted extract was completely loaded, the cartridges were washed with 5 mL of water and dried under vacuum, and the compounds were eluted using 9 mL of ACN. Subsequently, the extracts were evaporated to 1 mL in a nitrogen-flow TurboVap LV evaporator device (Caliper Life Sciences, Hopkinton, MA, USA). Aliquots of 125 µL were reconstituted in 250 µL of 50:50 (v:v) ACN:oxalic acid (aq., 0.01 mol·L^−1^, pH 2) and filtered through 0.22-µm polypropylene filters (Clarify-PP, Phenomenex, Torrance, CA, USA) before UHPLC-MS/MS analysis.

Considering the higher content of organic matter in manure compared to soil, 500-mg Oasis HLB cartridges were used for the clean-up step in this case, and the sample loading volume (1 mL diluted to 20 mL with citrate buffer (0.05 mol·L^−1^, pH 4) or 2 mL diluted to 40 mL) was evaluated. Under optimal conditions, 2 mL manure extract diluted to 40 mL with the citrate buffer (0.05 mol·L^−1^, pH 4) were loaded onto 500 mg Oasis HLB cartridges, which were previously conditioned with 10 mL of ACN, 10 mL of Milli-Q water and 10 mL of citrate buffer. The subsequent elution, evaporation and reconstitution steps were performed as previously explained for soil:compost samples.

### UHPLC-MS/MS analysis

The chromatographic separation was performed as in a previous work done by the research group [33]. Briefly, an Agilent 1290 Infinity II UHPLC device (Agilent Technologies) was used with a Kinetex C_18_ polar 100 Å (2.1 × 5 mm, 2.6 μm) precolumn and a Kinetex C_18_ polar 100 Å (2.1 × 50 mm, 2.6 μm) column (Phenomenex, Alcobendas, Spain). An Agilent 6430 Triple Quad tandem mass-spectrometer (QqQ) by Agilent Technologies was used as mass analyser, and the quantification was carried out in dynamic multiple reaction monitoring (DMRM) acquisition mode. The electrospray ionisation source worked in the positive mode (ESI +) for all the analytes. Fragmentor voltages (V), collision energies (eV) and parent and product ions (m/z) for the target analytes and surrogates are summarised in Table S2.

### QA/QC and material cleaning procedure

In order to overcome background contamination problems that could derived in false positives detection, all the material employed during the assays was thoroughly cleaned. Briefly, non-reused glass test tubes were heated at 350 °C for an hour in a muffle furnace (HD-230, Hobersal, Caldes de Monbui, Catalonia, Spain). Then, they were washed with an aqueous solution of 5% (w/w) disodium ethylenediaminetetraacetate dihydrate (Na_2_EDTA, 100%, PanReac AppliChem) in an ultrasound bath (J. P. SELECTA, Abrera, Catalonia, Spain) in order to avoid the possible interaction of the target analytes with the silanol groups on the glass surface [35, 39, 40]. The clean-tubes were rinsed and ultrasonicated with Milli-Q water and dried at 100 °C. Ceramic homogenizers were ultrasonicated first with Milli-Q water and then with dichloromethane. Eventually, they were heated at 350 °C for an hour before use. Regarding plastic material, it was all submerged in a 10% HNO_3_ (69%, Merck) bath for 48 h, rinsed with Milli-Q water and dried at 50 °C before use.

Validation of the method according to the criteria described in the Eurachem Guide [41] and the Regulation (EU) 2021/808 [42] was assessed in terms of absolute and apparent recovery, precision in terms of repeatability and instrumental and procedural limits of quantification. Chromatographic identification of the target analytes in the samples was performed according to the identification criteria established in the Council Directive 96/23/EC [43]. The presence of the compound was confirmed by the comparison of the retention time with a reference standard as well as the presence of the two most specific transitions of each target compound. Matrix effect at the detection for both solid samples was assessed using extracts spiked with the target compounds just before the chromatographic analysis.. Matrix-matched calibration and the use of labelled compounds as surrogate approaches were used for correction of absolute recoveries and apparent recovery calculations. Although both strategies were applied for all the analytes included in this method, the optimal strategy is understood as the one that gives values between 70 and 130% in each case. Regarding the surrogate selection, a labelled analogue for each antimicrobial family was selected.

Lastly, in each optimisation and sample analysis experiments, sets at least three blank samples (with and/or without matrix) were included and treated using the same analytical procedure used for spiked samples.

### UHPLC-q-Orbitrap analysis and suspect screening

Soil and manure samples were also analysed through a Thermo Scientific Dionex UltiMate 3000 UHPLC coupled to a Thermo Scientific Q Exactive Focus quadrupole-Orbitrap mass spectrometer (UHPLC-q-Orbitrap) equipped with a heated ESI source (HESI, Thermo-Fisher Scientific, CA, USA) in order to extent the multitarget method to suspect screening of more than 22,278 suspects. Briefly, extracts were injected on an ACE UltraCore XB-C_18_ (2.1 mm × 150 mm, 1.7 µm) chromatographic column with a pre-filter (2.1 mm ID, 0.2 µm) from Phenomenex. Concerning the mobile phase, Milli-Q water (A) and MeOH (B), both containing 0.1% HCOOH, were used in positive ionisation mode. Flow rate was set to 0.3 mL·min^−1^, column temperature at 50 °C and 5 µL were injected maintaining the autosampler at 5 °C. All the samples were injected in triplicate. The q-Orbitrap operated in full scan-data dependent MS2 (full MS-ddMS2) discovery acquisition mode. The intensity threshold and dynamic exclusion for the data dependent were respectively 8.0 × 10^3^ and automatically set up. The scan range was *m/z* 70–1050, the Full MS had a resolution of 70,000 FWHM for a 200 m*/z* relation, and it was followed by three ddMS2 scans with a resolution of 17500 FWHM with an isolation window of 3 m*/z*. The stepped normalised collision energy (NCE) in the higher-energy collision dissociation (HCD) cell was set at 10–30-70 eV, and the MS2 was a sum of the fragmentations obtained with the different energies. HESI source parameters were set to 3.8 kV for the spray voltage, 360 °C for the capillary temperature, 40 arbitrary units (au) for the sheath gas (nitrogen), 15 au for the auxiliary gas and 310 °C for the auxiliary gas heater, and S-lens RF level was set at 55.0. Pierce LTQ ESI Calibration Solutions (Thermo-Fisher Scientific) were used for external calibration of the instrument every 3 days. Xcalibur 4.0 (Thermo-Fisher-Scientific) was used for controlling the chromatographic and mass spectrometric systems.

For suspect screening, the Compound Discoverer 3.3 (Thermo-Fisher Scientific) software was used. Regarding the peak picking criteria, only features with a minimum peak area of 500,000 and a Lorentzian peak shape were manually picked for further annotation. Only exact masses with an error lower than ± 5 ppm were considered using nine different mass lists, being five of them metabolic transformation products obtained by BioTransformer 3.0 software (19,483 compounds) and the other four including antimicrobials from different families as well as TPs (2795 compounds), summing a total of 22,278 compounds. The molecular formulas suggested by Compound Discoverer were considered for annotation if MS1 spectra was satisfactorily matched (SFit > 30% and isotopic profile > 80%), and a minimum peak to consider of 5e^5^ units was established. Furthermore, group (injection replicates) coefficient of variation (CV) less or equal to 30% in any sample group, peaks 10 times larger than the blanks and with a relative standard deviation (RSD %) lower than 30% within injection replicates were taken into account. Moreover, it was limited to molecules containing O, N, Cl, Br, S and/or F due to the specific antimicrobial molecules containing those atoms. Noise elimination was performed using ACN blanks as reference, and only those features included in the Mass Lists were considered. When MS2 was available, it was compared with the corresponding spectra in mzCloud database (https://www.mzcloud.org/), and a threshold value of 70% was considered for positive identification. Fragmentation was also evaluated with the mzLogic tool (Thermo-Fisher Scientific), and a threshold value of minimum 60% was also considered. Retention times were estimated from the Retention Time Index (RTI) platform (http://rti.chem.uoa.gr/), and the candidates were rejected or accepted depending on whether or not there was a statistical difference with the estimated value within the uncertainty of the built model (only box 1 and box 2 candidates were considered). Confirmed candidates were assigned using Schymanski [44] scale from 1 to 3 levels of identification. In level 3, evidence exists for providing possible structures, but insufficient information for one exact structure only (e.g. positional isomers), and hence, tentative candidates were proposed. On the contrary, in levels 1 and 2, the structure is identified. The difference between them is that in level 1, the structure is confirmed via appropriate measurement of a reference standard with MS, MS^2^ and retention time matching, whereas in level 2, probable structure is proposed using different evidence such as MS^2^ library matching (level 2a) or diagnostic MS^2^ in silico fragmentation (level 2b) when no standard or experimental MS^2^ database is available.

## Results and discussion

### Injection solvent

For the injection solvent choice, a previous study based on the chromatographic analysis of SAs and TCs was taken as reference [33]. In that work, different organic solvents (ACN and MeOH) and aqueous mixtures of them were evaluated as injection solvents, resulting ACN:oxalic acid (50:50, v:v) (aq., 0.01 mol·L^−1^, pH 2) the best alternative in terms of chromatographic resolution and TCs’ epimerization avoidance. Hence, ACN:oxalic acid mixture was tested for the analysis of the rest of the antimicrobials included in this work. Figure S1–S4 showed the chromatograms for a representative antimicrobial of each group in the evaluated injection solvent. As it can be seen, ACN:oxalic acid mixture retrieved adequate resolution and peak symmetry for the target antimicrobials, and thus, it was used in further experiments.

### Extraction optimisation

#### FUSLE vs QuEChERS

**Fig. 1:**
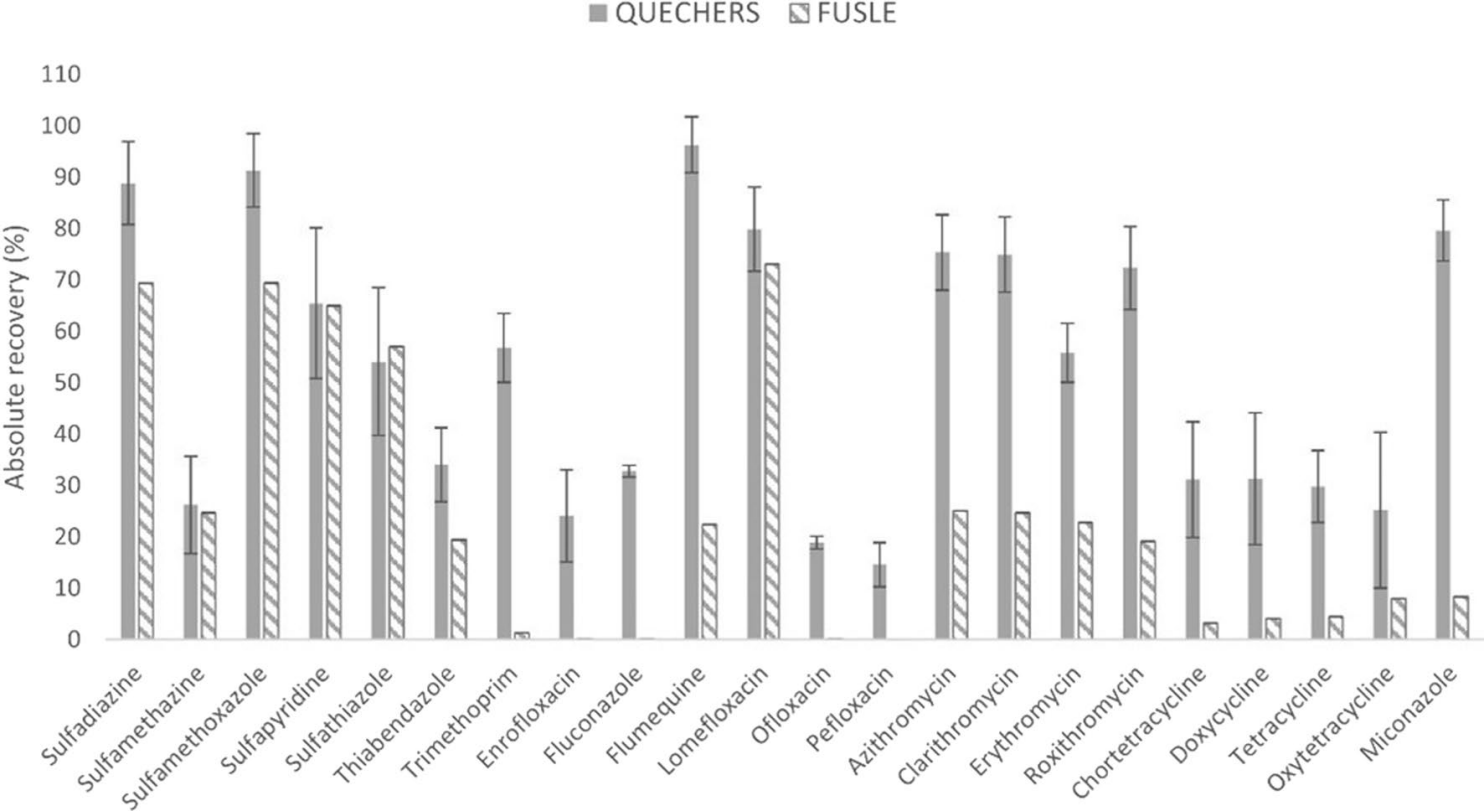
Absolute recoveries for each target analyte (*n* = 4) after performing QuEChERS-SPE (quick, easy, cheap, efficient, rugged and safe solid-phase extraction) or FUSLE-SPE (focused ultrasound solid–liquid extraction-solid phase extraction) extraction (data not available for danofloxacin and mycophenolic acid)

The adequacy of FUSLE and QuEChERS (at pH 2.5) was assessed in terms of the number of analytes recovered in each protocol and their absolute recovery values. For that assessment, spiked (*n* = 4) soil:compost samples and non-spiked (*n* = 4) soil:compost samples were extracted using both approaches, and the extracts (diluted to 70 mL and adjusted at pH 4 with the citrate buffer) were cleaned-up using SPE. Results are summarised in Fig. 1.

The QuEChERS method enabled the extraction of almost all the antimicrobials from the soil:compost samples with absolute recovery values between 24 and 91% for twenty out of twenty-two analytes evaluated (danofloxacin and mycophenolic acid were not available in the lab during this optimisation assay, but they were later included in the validation). Lower absolute recoveries were retrieved for ofloxacin and pefloxacin, 19% and 15%, respectively. Adequate repeatability was obtained with RSD values below 15% for all the target compounds. Regarding FUSLE extraction, only ten out of twenty-two analytes could be recovered in the range of 22–73%. Comparing with QuEChERS-based extraction, no statistical differences were observed for the extraction of SAs *(t* = 0.6 < *t*_crit_ = 2.3), but the recoveries for TCs, MCs, thiabendazole, trimethoprim and miconazole decreased significantly, and the extraction of FQs was neither improved using FUSLE. Furthermore, RSD values higher than 57% were obtained using FUSLE (not included in Fig. 1 to avoid scale issues), showing its inadequacy in terms of precision. For all this, QuEChERS-based extraction was chosen as optimal and used in further experiments.

#### Assessment of samples’ pH in QuEChERS

**Fig. 2:**
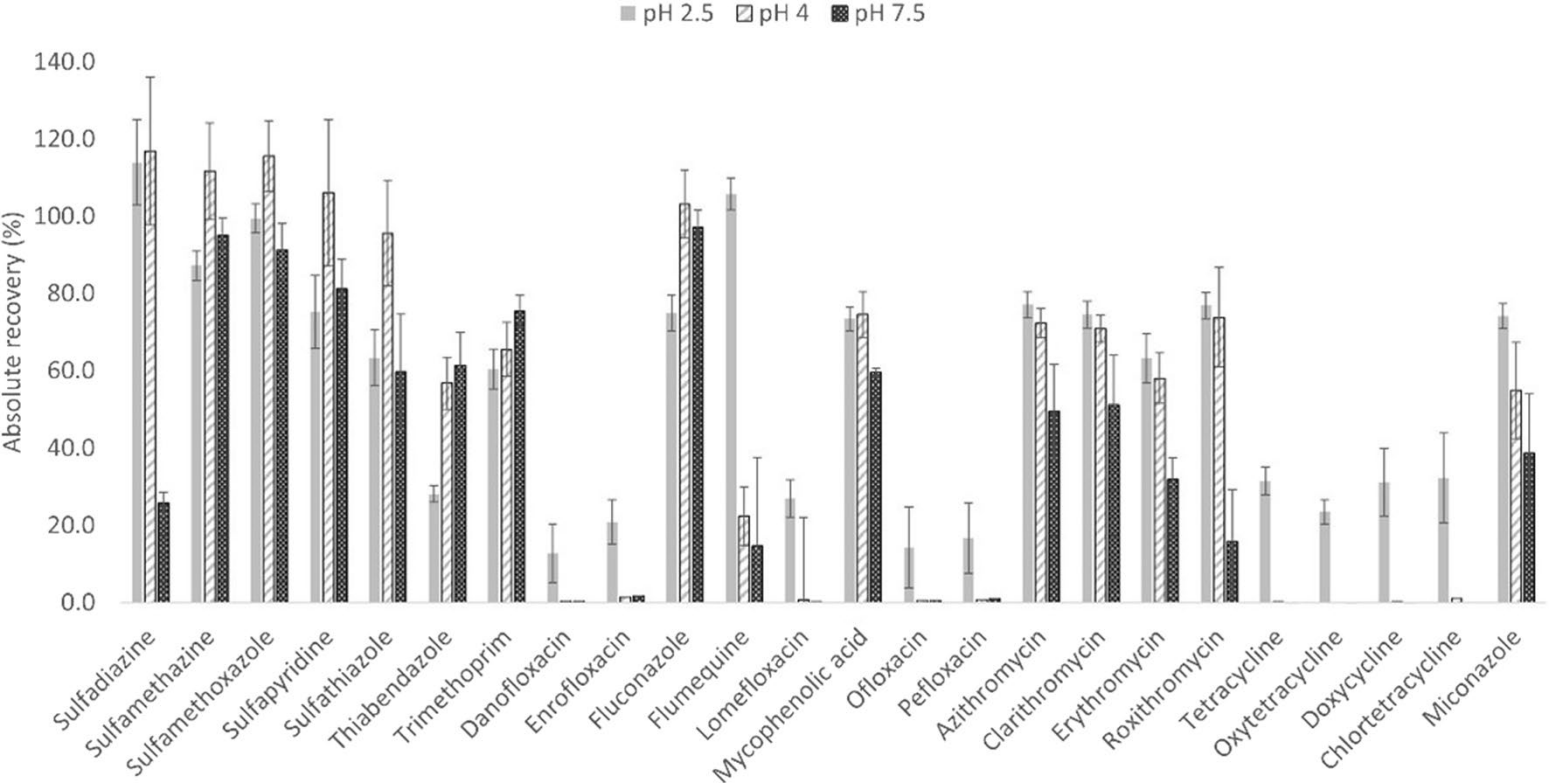
Absolute recoveries (*n* = 3) of the target analytes in QuEChERS (quick, easy, cheap, efficient, rugged and safe) performed at the different pH values tested (data for danofloxacin and mycophenolic acid included)

Sample was adjusted at different pH values (i.e. pH 2.5, pH 4 and pH 7.5) and the QuEChERS extraction absolute recovery was determined (see Fig. 2). According to the results, pH 7.5 rendered the lowest recoveries among the studied pH values. Although extractions performed at pH 4 provided slightly higher extraction recoveries for some SAs (96–117% at pH 4 vs 63–114% at pH 2.5) and fluconazole (103% at pH 4 vs 75% at pH 2.5), TCs and FQs were not extracted under those extraction conditions. Therefore, pH 2.5 was set as the optimal value for extraction using QuEChERS, with only three (danofloxacin, ofloxacin and pefloxacin) out of twenty-four analytes showing absolute recoveries values under the 20%.

### Optimisation of SPE clean-up step

The amount of the SPE sorbent together with the extract loading volume (70 mL in 200 mg cartridges or 140 mL in 500 mg cartridges) and pH (pH 4 or pH 7.5) were evaluated in triplicate using spiked soil:compost samples extracted with QuEChERS (at pH 2.5) (see the “QuEChERS” section).

**Fig. 3:**
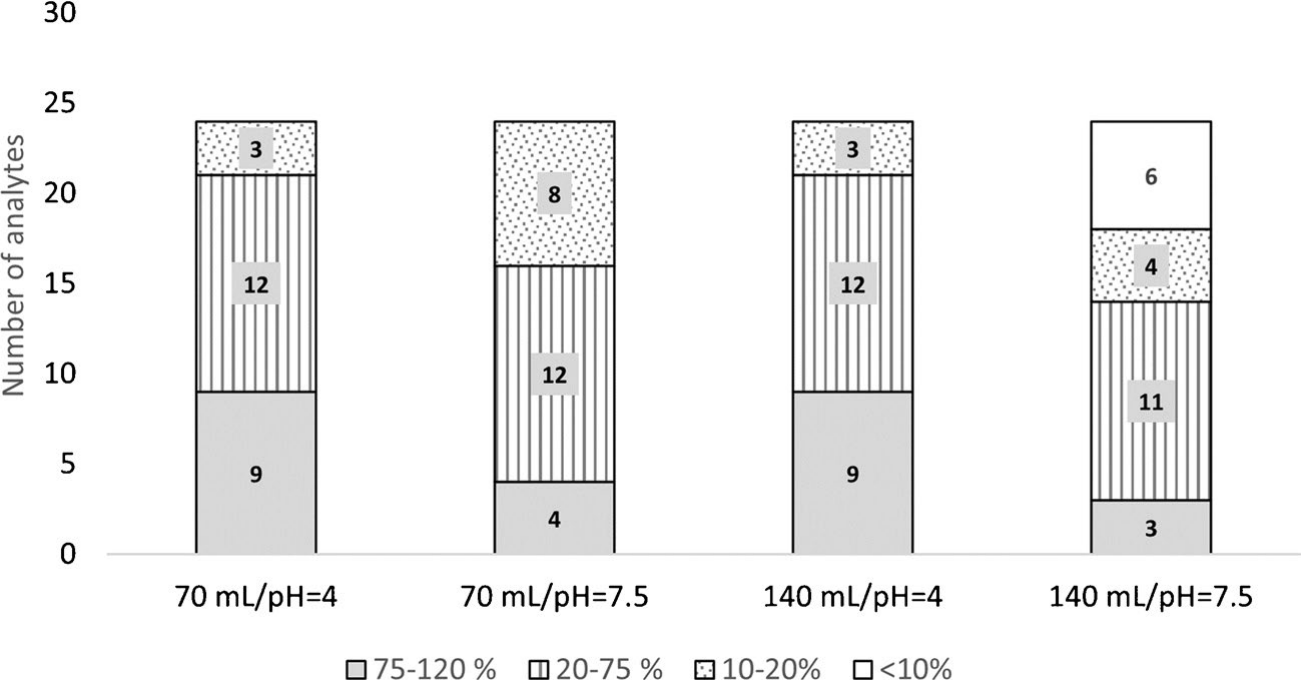
Absolute recovery values and number of analytes recovered in soil:compost samples in each assessed condition in the clean-up step: sample pH (pH 4 and pH 7) and sample loading volumes (70 mL and 140 mL)

The studied pH values were selected according to the acidic and basic properties of FQs (Table S1). Above pH 4, the functional groups can be ionised with both positive and negative charges. Ion compensation leads to an electrically neutral compound which tend to show a better absorption in the Oasis HLB SPE cartridges [18]. According to the observations (see Fig. 3), different extraction recoveries were obtained depending on the extracts’ pH. Assays performed at pH 4 rendered higher extraction recoveries for TCs, regardless of the loading extract volume, and for MCs, especially when large extract volumes (140 mL) were loaded (*t* = 7.4 > *t*_crit_ = 2.5 for TCs and *t* = 9.1 > *t*_crit_ = 2.5 for MCs). No statistical differences (*t* = 0.1 < *t*_crit_ = 2.0) were observed in the assays performed at pH 4 and different loading volumes/cartridge mass (i.e. 70 mL onto 200 mg-cartridges and 140 mL onto 500 mg cartridges). Based on the previous results, and taking into account that a higher dilution of the extract (and, hence, larger loading-time) was required in the case of 140 mL volume in order to guarantee an effective loading in SPE (< 5% (v/v) of organic solvent in the loading solution), 70 mL was set as optimum loading volume.

**Fig. 4:**
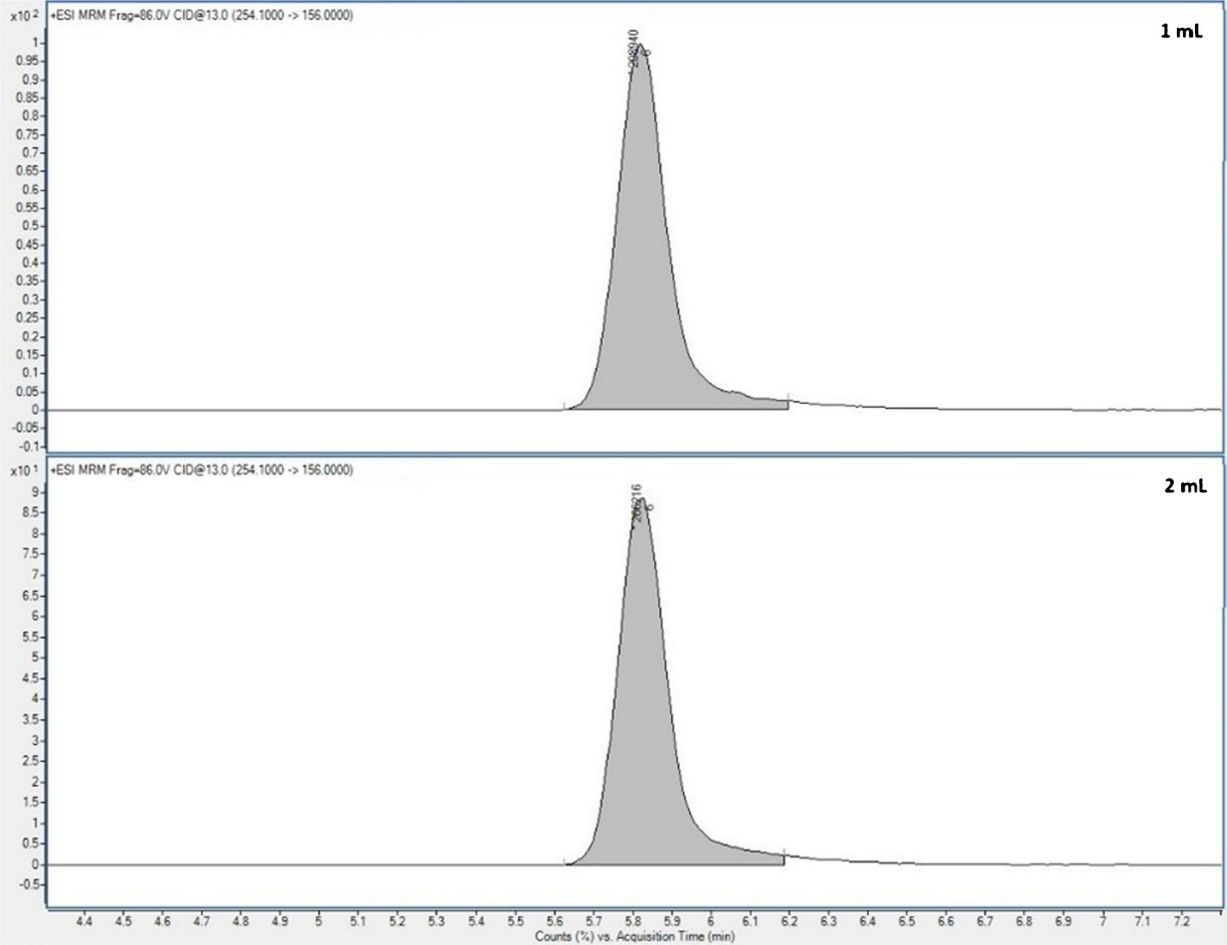
Chromatograms for sulfamethoxazole (as representative of the target analytes) in manure samples with loading volumes to the 500 mg-SPE cartridges of 20 mL or 40 mL

The optimum clean-up SPE conditions established for soil:compost samples were used for the clean-up of manure extracts, but using 500 mg-Oasis HLB-cartridges; thus, the extract loading volume was studied. No statistical differences were observed in the chromatographic peak symmetry, in the retention times (see Fig. 4), nor in the extraction efficiencies (*t* = 0.1 < *t*_crit_ = 2.0) when loading 20 mL or 40 mL of the manure extracts to the SPE cartridges. Therefore, in order to achieve a higher preconcentration factor, an extract loading volume of 40 mL was established as optimal.

### Figures of merit

#### Instrumental parameters

Quantification of the target analytes was performed by a twelve-points external calibration curve (0.25–100 μg·kg^−1^) prepared in ACN:oxalic (50:50, v:v). Adequate linearity, with determination coefficients (*r*^2^) ranging from 0.997 to 0.999, was observed for each of the target analytes. Furthermore, instrumental repeatability and intermediate repeatability of the measurements with the UHPLC-MS/MS system were also assessed by injecting in triplicate the external calibration solutions in the range of 0.25–25 μg·kg^−1^ in the same day and different days, respectively. Adequate repeatability and intermediate repeatability (with RSD % values lower than 25% and 22%, respectively) were observed for all the analytes at all concentration levels. Furthermore, for each of the target analytes, the instrumental limits of quantification (LOQ_INS_) were calculated as the lowest external calibration point with a relative standard deviation (RSD %) and a systematic error in relation to the theoretical value below 30%. LOQ_INS_ were below 0.93 μg·kg^−1^ for all the compounds included in this work (Table S3).

#### Procedural limits of quantification (LOQ_PRO_)

LOQ_PRO_ were calculated from the sum of the average signal of the procedural blanks plus ten times their standard deviations. To that result, the external calibration and absolute recovery were applied, obtaining the final LOQ_PRO_ values for each analyte in the sample. In the case of soil:compost samples (see Table 1) LOQ_PRO_ ranged from 0.45 to 7.50 μg·kg^−1^. Pefloxacin was the only antimicrobial that could not be validated at the lowest level since the LOQ_PRO_ value was 9.89 μg·kg^−1^. Comparing with the values published by Meng et al. [27] (see Table S4), better LOQ_PRO_ values were obtained in this study for sulfadiazine, sulfathiazole, sulfapyridine and clarithromycin, whereas better values were reported for erythromycin and roxithromycin in that work. In the work published by Salvia et al. [45], lower LOQ_PRO_ values (0.02–2.14 ng·g^−1^) were reported, but the LOQ_PRO_ estimation was done in a different way in that work (i.e. as the analyte concentration that produced a peak signal of ten times the background noise from the chromatogram).

**Table 1:** Procedural limits of quantification (LOQ_PRO_), absolute and apparent recoveries and precision expressed as relative standard deviation (RSD %) at 10 µg·kg^−1^, 25 µg·kg^−1^ and 50 µg·kg^−1^ for soil:compost samples

Antimicrobials	LOQ_PRO_ (µg·kg^−1^)	10 µg·kg^−1^			25 µg·kg^−1^			50 µg·kg^−1^			Surrogate
		R%_abs_ (RSD%)	R%_surrog_ (RSD%)	R%_MM_ (RSD%)	R%_abs_ (RSD%)	R%_surrog_ (RSD%)	R%_MM_ (RSD%)	R%_abs_ (RSD%)	R%_surrog_ (RSD%)	R%_MM_ (RSD%)	
Sulfadiazine	1.31	61 (6)	106 (10)	128 (5)	53 (6)	92 (2)	109 (5)	53 (6)	101 (3)	110 (6)	[^2^H_4_]-sulfamethazine
Sulfamethazine	7.50	34 (10)	58 (5)	128 (< 1)	45 (4)	79 (5)	110 (3)	48 (5)	91 (3)	102 (5)	[^2^H_4_]-sulfamethazine
Sulfamethoxazole	2.71	89 (10)	107 (16)	120 (10)	79 (11)	100 (5)	105 (11)	79 (7)	107 (2)	104 (7)	[^13^C_6_]-sulfamethoxazole
Sulfapyridine	0.45	52 (5)	90 (8)	127 (4)	48 (7)	84 (4)	111 (6)	47 (4)	88 (4)	105 (4)	[^2^H_4_]-sulfamethazine
Sulfathiazole	2.05	51 (3)	89 (7)	137 (2)	44 (4)	77 (2)	116 (3)	42 (3)	79 (5)	109 (4)	[^2^H_4_]-sulfamethazine
Thiabendazole	4.67	25 (7)	131 (10)	119 (7)	23 (8)	128 (5)	105 (7)	22 (4)	116 (3)	99 (4)	[^2^H_5_]-enrofloxacin
Trimethoprim	1.11	42 (10)	72 (12)	127 (10)	38 (4)	66 (6)	119 (4)	33 (7)	63 (7)	104 (6)	[^2^H_4_]-sulfamethazine
Danofloxacin	5.77	9 (5)	48 (6)	137 (2)	7 (7)	43 (13)	100 (6)	8 (5)	43 (1)	100 (5)	[^2^H_5_]-enrofloxacin
Enrofloxacin	7.00	18 (11)	97 (14)	118 (10)	16 (7)	91 (1)	106 (7)	15 (4)	81 (3)	98 (4)	[^2^H_5_]-enrofloxacin
Fluconazole	2.40	79 (4)	101 (1)	116 (4)	73 (6)	91 (< 1)	102 (6)	68 (4)	92 (3)	93 (5)	[^13^C_6_]-sulfamethoxazole
Flumequine	2.60	73 (1)	94 (5)	94 (1)	70 (6)	88 (< 1)	93 (6)	68 (2)	93 (7)	93 (2)	[^13^C_6_]-sulfamethoxazole
Lomefloxacin	2.49	21 (11)	108 (6)	114 (10)	20 (1)	111 (6)	104 (1)	19 (4)	104 (4)	101 (4)	[^2^H_5_]-enrofloxacin
Mycophenolic acid	4.72	92 (1)	116 (27)	124 (32)	54 (8)	98 (5)	90 (8)	57 (10)	99 (11)	94 (10)	[^2^H_7_]-roxithromycin
Ofloxacin	6.95	12 (3)	66 (7)	130 (3)	11 (15)	60 (10)	105 (13)	11 (7)	56 (1)	99 (7)	[^2^H_5_]-enrofloxacin
Pefloxacin	9.89	11 (12)	58 (14)	105 (12)	11 (9)	63 (5)	109 (9)	11 (11)	57 (12)	104 (10)	[^2^H_5_]-enrofloxacin
Azithromycin	1.68	67 (9)	106 (4)	124 (8)	56 (2)	100 (3)	102 (2)	56 (5)	97 (1)	102 (5)	[^2^H_7_]-roxithromycin
Clarithromycin	1.25	71 (9)	111 (6)	127 (8)	57 (5)	102 (< 1)	101 (5)	57 (5)	99 (1)	102 (4)	[^2^H_7_]-roxithromycin
Erythromycin	4.51	48 (6)	76 (< 1)	99 (5)	50 (2)	90 (3)	94 (2)	49 (9)	85 (5)	90 (8)	[^2^H_7_]-roxithromycin
Roxithromycin	7.26	34 (15)	47 (19)	124 (8)	39 (8)	70 (3)	97 (5)	50 (4)	86 (2)	103 (3)	[^2^H_7_]-roxithromycin
Tetracycline	2.53	31 (2)	121 (12)	120 (3)	29 (4)	100 (4)	112 (4)	26 (2)	101 (12)	100 (2)	[^2^H_6_]-tetracycline
Oxytetracycline	2.69	26 (17)	91 (12)	127 (2)	22 (7)	90 (8)	111 (6)	22 (7)	91 (11)	109 (7)	[^2^H_6_]-tetracycline
Doxycycline	4.33	32 (3)	125 (13)	123 (2)	29 (1)	120 (13)	103 (1)	30 (6)	118 (16)	104 (6)	[^2^H_6_]-tetracycline
Chlortetracycline	1.98	39 (25)	132 (26)	94 (13)	36 (7)	138 (5)	106 (7)	33 (2)	130 (17)	99 (3)	[^2^H_6_]-tetracycline
Miconazole	0.73	60 (2)	95 (6)	126 (2)	52 (5)	95 (8)	108 (5)	51 (7)	88 (3)	105 (6)	[^2^H_7_]-roxithromycin

Regarding manure samples (see Table 2), all values were under 3.21 μg·kg^−1^ as exception of the FQs ofloxacin (5.53 μg·kg^−1^) and pefloxacin which could not be quantified by this method under 15.75 μg·kg^−1^, the same as in soil:compost samples. Similar values were released by Hou et al. [25] (see Table S4) for TCs and FQs even though they defined LOQ_PRO_ as the lowest concentration levels corresponding to a signal-to-noise (S/N) ratio of ten. Another scientific work, which defined the LOQ_PRO_ as equal to ten times the standard deviation of the results for a series of replicates used to determine a justifiable limit of detection (LOD), reported higher LOQ_PRO_ values in broiler manure (3.0–14.0 μg·kg^−1^) [46].

**Table 2:** Procedural limits of quantification (LOQ_PRO_), absolute and apparent recoveries and precision expressed as relative standard deviation (RSD %) at 10 µg·kg^−1^, 25 µg·kg^−1^ and 50 µg·kg^−1^ for manure samples

Antimicrobials	LOQ_PRO_ (µg·kg^−1^)	10 µg·kg^−1^			25 µg·kg^−1^			50 µg·kg^−1^			Surrogate
		R%_abs_ (RSD%)	R%_surrog_ (RSD%)	R%_MM_ (RSD%)	R%_abs_ (RSD%)	R%_surrog_ (RSD%)	R%_MM_ (RSD%)	R%_abs_ (RSD%)	R%_surrog_ (RSD%)	R%_MM_ (RSD%)	
Sulfadiazine	0.31	70 (10)	88 (5)	104 (10)	62 (10)	82 (7)	92 (10)	67 (3)	90 (4)	96 (3)	[^13^C_6_]-sulfamethoxazole
Sulfamethazine	0.31	57 (9)	102 (3)	98 (8)	55 (8)	101 (2)	94 (8)	58 (2)	103 (4)	102 (2)	[^2^H_4_]-sulfamethazine
Sulfamethoxazole	2.92	81 (7)	102 (1)	112 (7)	72 (10)	97 (5)	98 (9)	74(4)	100 (3)	89 (4)	[^13^C_6_]-sulfamethoxazole
Sulfapyridine	0.41	50 (2)	90 (5)	98 (2)	47 (8)	86 (2)	91 (8)	51 (2)	90 (4)	102 (2)	[^2^H_4_]-sulfamethazine
Sulfathiazole	1.03	35 (13)	63 (15)	103 (13)	25 (10)	47 (9)	74 (10)	34 (7)	61 (8)	97 (7)	[^2^H_4_]-sulfamethazine
Thiabendazole	0.93	36 (4)	92 (3)	114 (24)	21 (8)	89 (2)	77 (8)	23 (1)	80 (15)	73 (1)	[^2^H_5_]-enrofloxacin
Trimethoprim	1.98	13 (29)	49 (29)	75 (29)	11 (16)	47 (7)	72 (12)	17 (19)	50 (6)	91 (11)	[^2^H_5_]-enrofloxacin
Danofloxacin	2.93	15 (25)	44 (6)	121 (12)	14 (12)	58 (7)	80 (8)	23 (17)	67 (3)	79 (15)	[^2^H_5_]-enrofloxacin
Enrofloxacin	2.39	35 (22)	103 (1)	117 (1)	24 (8)	98 (3)	72 (1)	36 (18)	103 (5)	96 (18)	[^2^H_5_]-enrofloxacin
Fluconazole	0.94	62 (11)	81 (17)	91 (11)	66 (9)	96 (2)	96 (9)	68 (2)	95 (5)	109 (2)	[^2^H_7_]-roxithromycin
Flumequine	2.27	88 (5)	112 (4)	108 (5)	80 (9)	107 (5)	97 (9)	83 (6)	112 (1)	93 (6)	[^13^C_6_]-sulfamethoxazole
Lomefloxacin	1.51	27 (16)	81 (14)	108 (15)	23 (5)	96 (10)	91 (5)	25 (9)	74 (7)	92 (9)	[^2^H_5_]-enrofloxacin
Mycophenolic acid	2.10	74 (7)	97 (7)	109 (7)	66 (9)	97 (3)	96 (9)	69 (6)	97 (3)	92 (6)	[^2^H_7_]-roxithromycin
Ofloxacin	5.53	33 (20)	97 (7)	111 (18)	23 (9)	97 (4)	77 (9)	31 (17)	90 (4)	90 (17)	[^2^H_5_]-enrofloxacin
Pefloxacin	15.75	-	-	-	18 (15)	76 (10)	78 (14)	25 (20)	73 (7)	88 (20)	[^2^H_5_]-enrofloxacin
Azithromycin	0.77	68 (8)	89 (3)	105 (8)	62 (6)	90 (1)	94 (6)	66 (5)	92 (3)	95 (5)	[^2^H_7_]-roxithromycin
Clarithromycin	0.32	71 (8)	92 (1)	107 (8)	62 (7)	91 (1)	94 (7)	66 (6)	91 (1)	93 (6)	[^2^H_7_]-roxithromycin
Erythromycin	2.21	95 (16)	123 (9)	132 (16)	69 (9)	101 (4)	94 (8)	73 (8)	102 (1)	76 (8)	[^2^H_7_]-roxithromycin
Roxithromycin	1.99	74 (8)	97 (2)	109 (8)	66 (8)	96 (3)	95 (8)	70 (7)	97 (< 1)	92 (7)	[^2^H_7_]-roxithromycin
Tetracycline	2.65	39 (10)	112 (3)	108 (10)	34 (17)	118 (6)	94 (16)	37 (17)	116 (4)	93 (17)	[^2^H_6_]-tetracycline
Oxytetracycline	2.60	34 (12)	99 (9)	138 (12)	26 (15)	91 (8)	105 (15)	21 (2)	79 (11)	72 (26)	[^2^H_6_]-tetracycline
Doxycycline	3.21	38 (10)	109 (3)	111 (9)	32 (8)	112 (16)	92 (8)	35 (14)	111 (5)	90 (14)	[^2^H_6_]-tetracycline
Chlortetracycline	3.04	44 (15)	121 (5)	111 (14)	37 (11)	120 (4)	92 (11)	41 (9)	125 (4)	90 (9)	[^2^H_6_]-tetracycline
Miconazole	2.09	54 (12)	97 (9)	103 (12)	49 (6)	90 (1)	89 (6)	56 (8)	99 (9)	97 (8)	[^2^H_4_]-sulfamethazine

#### Matrix effect at the detection

Matrix effect is an important factor affecting method trueness, especially when ESI is used for the analysis of complex matrices, since other matrix components could act as interferences altering the ionisation of the target analytes. In this work, matrix effect was calculated according to the Eq. 1, where B is the peak area of the analyte in a reference standard sample and A is the chromatographic peak area of the analyte in a sample spiked just before the chromatographic analysis at the same concentration as the standard solution.

1 $$Matrix\;effect\;\left(ME\%\right)=\left(A/B-1\right)\;\times\;100$$

**Fig. 5:**
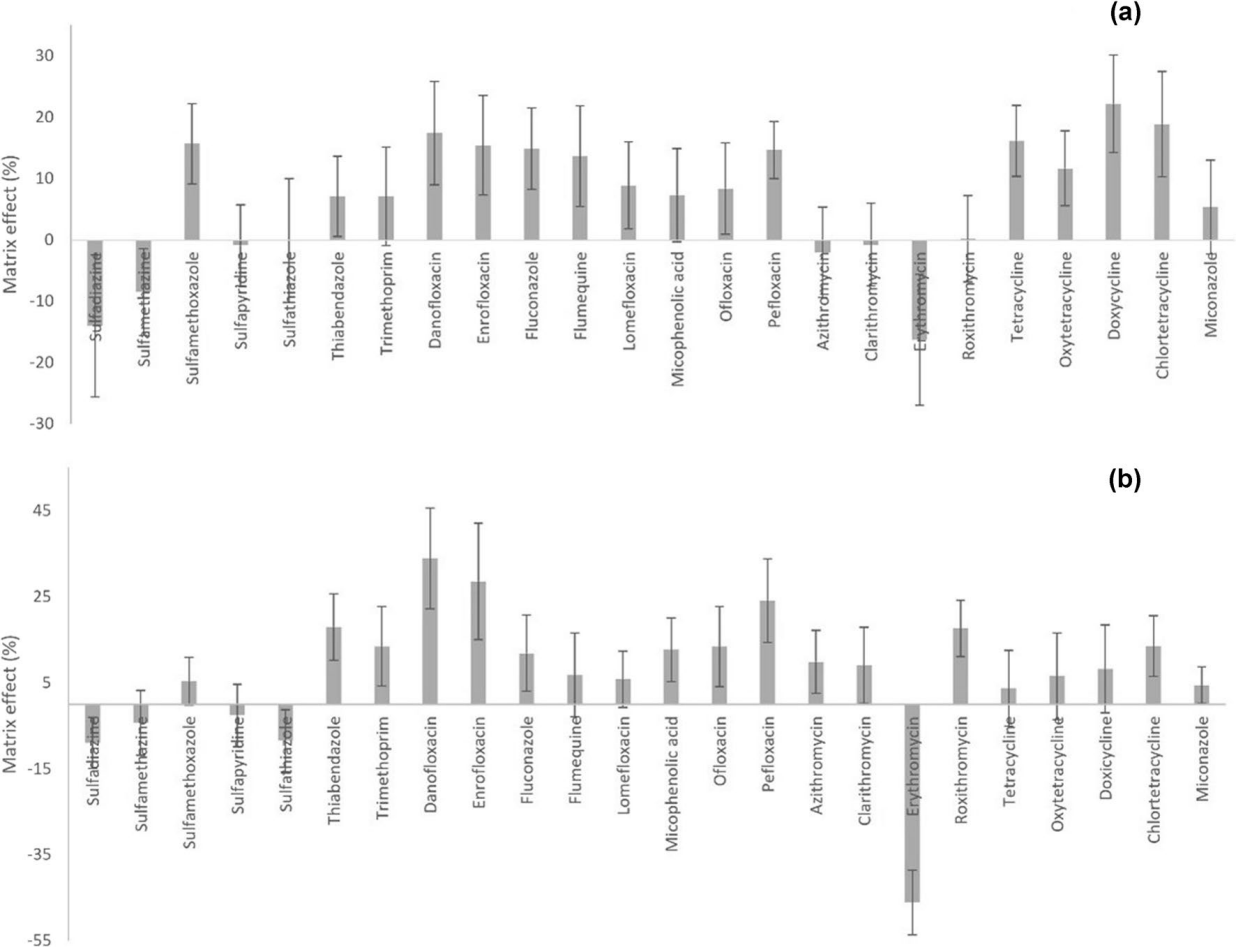
Matrix effect at the detection for each of the target analytes in soil:compost (**a**) and manure samples (**b**)

Negative values indicate a loss in the chromatographic signal (ion suppression) of the target analytes, which is the case of MCs and SAs, whereas positive values reflect an ion enhancement, as in the case of the rest of the antimicrobial families (see Fig. 5) in soil:compost samples. Overall, the matrix effect observed at detection was no so significant in this work, being all the values below 22%. The reason of the low matrix effect observed, regardless of the complex matrix analysed, could be the efficiency of the clean-up step. An SPE clean-up was also performed by Salvia et al. [45], using both a strong anion-exchange cartridge (SAX) and a polymeric cartridge (Strata-X); nevertheless, the sorbent choice for purification step seemed not to be the optimal as ion suppression over the − 30% was observed for SAs, whereas an enhancement of 242% was determined for roxithromycin at 50 μg·kg^−1^ validation level. Comparing with the results obtained by Meng et al. [27], this work retrieved significantly lower matrix effect for erythromycin, roxithromycin and sulfapyridine. In the work performed by Meng et al., the clean-up was performed by dispersive SPE (dSPE), in which the smaller amount of sorbent used (25 mg PSA + 10 g C_18_ vs 200 mg Oasis HLB cartridge) may result in less retention of co-eluting matrix interferences, generating a greater matrix effect in the detection. However, similar matrix effects was observed for some SAs in this work in comparison to other works using dSPE as clean-up strategy [23, 34].

Referring to animal manure, overall, an ion enhancement was observed for almost all the analytes, except for erythromycin, which showed the most significant negative ME % (− 46%). Anyway, for the rest of the target analytes, ME % remained under 30%. Even if Hou et al. employed SAX cartridges (3 mL/200 mg) followed by Oasis HLB cartridges (6 mL/500 mg) for manure sample purification, a higher matrix effect was observed for all the studied FQs, SAs, TCs, MCs and trimethoprim in comparison to the results obtained in this work [25].

#### Trueness and precision

The method including QuEChERS extraction was validated at three concentration levels (10, 25 and 50 μg·kg^−1^) in soil:compost and manure samples. Absolute recoveries were calculated using the external calibration curve. In order to determine the trueness of the method apparent recoveries were calculated using two different approaches: (i) the use of deuterated analogues as surrogates, which were spiked at the beginning of the procedure (ratio between the absolute recoveries of the target analytes and the corresponding surrogate compound) and (ii) using a six-point matrix-matched calibration (1–75 μg·kg^−1^). Before using this last approach to get concentrations, the adequacy of the calibration curves was assured (*r*^2^ between 0.975 and 0.999), and repeatability, expressed as RSD, less than 20%).

In the case of soil:compost samples, absolute recoveries (*n* = 3) ranged from 21 to 89% for 10 μg·kg^−1^ concentration level, from 20 to 79% for 25 μg·kg^−1^ and from 19 to 79% for 50 μg·kg^−1^ (see Table 1). In the three validation levels, lower recoveries (10–20%) were estimated for some FQs. These values were comparable to the ones determined by Salvia et al. [45] for SAs, although this method still achieved higher extraction efficiency for erythromycin. After performing a PLE-based extraction, da Silva et al. [21] reported higher recoveries for SAs, while similar values were obtained for TCs. Adequate apparent recoveries were obtained after surrogate correction for all the target compounds at the three tested levels, between 72–132%, 77–138% and 79–130% for low, medium and high concentration levels, respectively. The exception was found for sulfamethazine (58%) and roxithromycin (47%) antimicrobials at the lowest spiked concentration level (i.e. 10 μg·kg^−1^) and the three FQs danofloxacin, ofloxacin and pefloxacin with recoveries ranged from 43 to 66% in the three spiked levels. Even though those analytes fell outside the established ranged by the guideline 2021/808 [42], according to the standard SANTE/11813/2017, lower recoveries values (30–70%) are acceptable in case of their proven consistency (RSD < 20%). Precision, determined in terms of repeatability, at all concentration levels tested and expressed as RSD %, was lower than 20% for all the antimicrobials in all the validation points except for chlortetracycline and mycophenolic acid in 10 μg·kg^−1^ (26% and 23%, respectively), which means that the method was still reproducible and reliable for the compounds’ analysis at the studied concentration levels.

When matrix-matched calibration approach was used to determine apparent recoveries, all the antimicrobials included in this work fulfilled the trueness requirements (80–120%) established in the Regulation (EU) 2021/808 [42] at 25 μg·kg^−1^ and 50 μg·kg^−1^ levels. At the lowest validated concentration level (i.e. 10 μg·kg^−1^), the recovery upper limit went to 137%, but still with RSDs < 20%, except for mycophenolic acid (32%).

Comparing with the literature (see Table S4), the apparent recovery values obtained for soil:compost samples spiked at 50 μg·kg^−1^ were similar to the ones reported by Meng et al. [27], where QuEChERS-based analytical method was also used, for erythromycin, roxithromycin, clarithromycin, sulfamethazine and sulfamethoxazole, but the method proposed in this work provided better repeatability. Moreover, this work retrieved better results for sulfadiazine and sulfathiazole. Furthermore, this work also offered a higher accuracy for SAs (46–63% vs 105–116%) and flumequine (34% vs 93%) analysis than the method developed by Martínez-Piernas et al. [23], but similar recoveries were retrieved for trimethoprim and MCs. In the QuEChERS method developed by Lee et al. [34], lower recoveries were determined for SAs (65–73%) in comparison to the ones reported in our study.

Regarding manure samples (Table 2), overall, 21–95% absolute recovery values (*n* = 3) were obtained for all the analytes at the three evaluated validation levels, except in the cases of trimethoprim (11–17%) and danofloxacin (14–23%). RSD % remained below 20% indicating good precision, except for three FQs and trimethoprim at the lowest validation level (RSD 20–29%). Overall, when surrogate correction was applied, almost all antimicrobials accomplished the trueness criteria [43] retrieving recoveries inside 80–120% range. Only sulfathiazole, trimethoprim and danofloxacin showed recoveries of 44–67% indicating that a better surrogate selection should be done to fit the accuracy requirements for analysing those compounds with this method. Similar recovery values were reported by Berendsen et al. for some TCs, FQs, and erythromycin [47]. Higher trueness at the low and high validation levels has been reported in this work for TCs, FQs, SAs and MCs quantification in comparison to the work performed by Hou et al. [25]; nevertheless, they retrieved better recovery values for trimethoprim after surrogate correction (73% vs 49% at 10 μg·kg^−1^ validation level and 72% vs 50% at 50 μg·kg^−1^). Ho et al. [46] also obtained a better trimethoprim extraction at 200 μg·kg^−1^ validation level; nevertheless, this method still demonstrated to be more accurate for sulfadiazine and doxycycline quantification. However, when matrix-matched calibration strategy was used in this work, acceptable apparent recoveries were obtained, especially for those compounds that could not be corrected by the use of surrogates (i.e. the recoveries for sulfathiazole, trimethoprim and danofloxacin ranged from 72 to 121%).

## Method application 

### Target analysis

Although most of the analysed antimicrobials were detected bellow the LOQ_PRO_ (see Table 3) among the analysed soil samples, remarkable concentrations were determined for sulfamethazine (7.9 ± 0.8 µg·kg^−1^) and danofloxacin (27.1 ± 1.4 µg·kg^−1^) in Bizkaia and trimethoprim in Nafarroa (4.9 ± 0.5 µg·kg^−1^). The occurrence of sulfamethazine has also been reported by Wei et al. [30] in animal manure-amended soils in China at an average concentration of 15.6 µg·kg^−1^, whereas trimethoprim has also been detected by Ho et al. at similar concentrations in broiler manure-amended soils [48]. Trimethoprim was also detected in the plant samples collected in “Araba_1” (3.0 ± 0.4 µg·kg^−1^). The presence of thiabendazole and enrofloxacin was also observed at concentrations ≤ 3.0 µg·kg^−1^, although the highest concentration was detected for tetracycline in the “Nafarroa_2” sample, 56.8 ± 2.8 µg·kg^−1^ (Table 3).

**Table 3:** Detected antimicrobial concentrations (µg·kg^**−**1^) ± standard deviation (*n* = 3, 2 s 95% confidence level) in soil, manure and plant samples

Soil samples								
Antimicrobials/sampling sites	Nafarroa_3	Nafarroa_4	Nafarroa_5	Nafarroa_6	Araba_1	Araba_2	Bizkaia_1	Bizkaia_3
Sulfamethazine	< LOQ_PRO_	< LOQ_PRO_	< LOQ_PRO_	< LOQ_PRO_	n.d	n.d	7.9 ± 0.8	n.d
Trimethoprim	n.d	4.9 ± 0.5	n.d	n.d	< LOQ_PRO_	< LOQ_PRO_	n.d	n.d
Fluconazole	n.d	n.d	n.d	n.d	< LOQ_PRO_	n.d	n.d	n.d
Danofloxacin	n.d	n.d	n.d	n.d	n.d	n.d	n.d	27.1 ± 1.4
Plant samples								
Antimicrobials/sampling sites	Nafarroa_2	Nafarroa_6	Araba_1	Araba_3	Araba_5	Gipuzkoa_1	Gipuzkoa_2	
Sulfadiazine	n.d	< LOQ_PRO_	n.d	n.d	n.d	n.d	n.d	
Thiabendazole	n.d	n.d	n.d	n.d	n.d	n.d	2.4 ± 0.1	
Trimethoprim	n.d	n.d	3.0 ± 0.4	n.d	n.d	n.d	n.d	
Enrofloxacin	n.d	n.d	3.4 ± 0.1	n.d	n.d	n.d	n.d	
Tetracycline	56.8 ± 2.8	n.d	n.d	< LOQ_PRO_	n.d	< LOQ_PRO_	n.d	
Manure samples								
Antimicrobials/sampling sites	Nafarroa_1	Araba_1	Araba_2	Bizkaia_1	Bizkaia_2	Gipuzkoa_1	Gipuzkoa_2	
Sulfadiazine	n.d	n.d	n.d	5.2 ± 0.1	13.0 ± 0.8	n.d	n.d	
Sulfamethazine	45.0 ± 2.2	1.7 ± 0.3	2.9 ± 0.6	n.d	n.d	2.3 ± 0.1	1.9 ± 0.1	
Chlortetracycline	n.d	n.d	n.d	6.0 ± 0.2	n.d	10.7 ± 0.8	93.3 ± 6.8	
Oxytetracycline	9.3 ± 0.6	n.d	3.5 ± 0.9	n.d	n.d	n.d	n.d	
Enrofloxacin	n.d	n.d	n.d	4.1 ± 1.0	n.d	n.d	n.d	

*n.d.* not detected

Overall, antimicrobials were detected at higher concentration levels in sheep manure samples in comparison to soil and plant samples, whereas no antimicrobials were detected in horse manure samples. Concretely, SAs, TCs and FQs in a range of 1.7 ± 0.3 to 93.3 ± 6.8 µg·kg^−1^ were determined in sheep manure samples (see Table 3). The occurrence of the same antimicrobial groups in animal manure has been reported by Van Epps et al. [8], Hou et al. [25] and Berendsen et al. [47] in a concentration order of TCs > FQs > SAs, as in the present work. Ho et al. [48] also confirmed the presence of sulfadiazine and enrofloxacin in broiler manure.

The higher detection of antimicrobials in animal manure in comparison to soils and plants is consistent with what it has been observed in the literature. Moreover, the main three antimicrobial groups detected in the analysed samples (TCs, FQs and SAs) are also the ones frequently reported in the literature [8, 25, 47, 48] as they are the most employed pharmaceuticals in agriculture. As regards to the obtained concentration of each antimicrobial group, the high sorption coefficient values (K_d_ — the parameter used to estimate the sorptive exchange of chemicals between a water phase and a solid phase. It is defined as the ratio between the concentration of a compound in the sorbent and in the water, once the equilibrium has been reached [16, 49]) and strong adsorption onto soil particles of TCs and FQs make them more stable in soils and difficult their migration, leading to the detection of higher concentrations in comparison with SAs. SAs, with lower K_d_ values, are more prone to move down from the surface soils [50]. Even if the detected antimicrobial concentrations do not exceed the threshold established by the European Agency for the Evaluation of Medicinal Products[51], antimicrobials that are considered “critically” (FQs) or “highly” important (SAs and TCs) to human medicine by the WHO [29] were detected, being agricultural activities the most likely source of contamination.

### Suspect screening analysis

The optimised method for multitarget analysis was extended to monitor as many as possible antimicrobials in the analysed soil and manure samples using a suspect screening approach.

An initial quantity of 55,418 features was provided by the workflow applied and after filters application (see the “QA/QC and material cleaning procedure” section) the total amount of unknown features was reduced to 1085. Subsequently, the peak picking was performed manually leaving 61 features, which fulfilled the peak shape constrains, to check if found in the samples. The identified features together with their corresponding identification level are gathered in Table 4, being especially remarkable the identification of formyl-sulfamethazine (a specific TP of the antimicrobial sulfamethazine) at 2a Level in “Nafarroa 1” manure sample, where sulfamethazine was detected at the highest concentration** (**45.0 ± 2.2 µg·kg^−1^). Formyl-sulfamethazine has previously been identified as one of the TPs derived from the degradation of sulfamethazine by the fungus *Trametes versicolor* in some in vivo experiments performed in aqueous medium (pH 4.5) [52]. In concordance with what it has been observed in the targeted analysis, a higher number of identified features was detected in manure samples compared to soils, being the manure samples collected in “Bizkaia 2″ and”Araba 1″ locations the ones with the highest presence of antimicrobials. However, propiconazole, an antifungal identified at level 2a, was only detected in soil samples. The presence of this compound has been previously reported in sediments and water samples [53, 54]. Moreover, 5-azulenemethanol (level 2b) was the compound detected in the largest number of samples, including some soil samples from Nafarroa, Araba and Bizkaia and all the analysed sheep manure samples.

**Table 4:** Name, uses, molecular formula, exact mass, experimental retention time and confidence level of the identified features in the suspect screening

Feature	Name	Use	Formula	Mass error (ppm)	m/z	mzCloud Best Match	Spectral match (%) by in silico fragmentation	Smiles	tR_exp_ [min]	Confidence of RTI^i^ (box)	Confidence level^ii^	Samples
1	Piperonylonitrile	Alter the growth of Nif + and Nif — strains of *K. pneumoniae*. Toxic against *D. farinae*, *D. pteronyssinus*, and *T. putrescentiae*	C8 H5 N O2	0.30	148.03935	83.3	-	N#CC1 = CC2 = C(OCO2)C = C1	4.978	1	2a	Manure: Nafarroa 1, Gipuzkoa 1 & 2, Bizkaia 1 & 2, Araba 1 & 2
2	Butyl 4-aminobenzoate	Antibacterial	C11 H15 N O2	0.84	194.11772	68.5	-	CCCCOC(= O)C1 = CC = C(N)C = C1	10.325	2	2a	Manure: Gipuzkoa 1 & 2, Bizkaia 1 & 2, Araba 1 & 2
3	Indirubin	Anti-inflammatory, antibacterial, detoxification, enhancing immune function anticancer	C16 H10 N2 O2	− 0.39	263.0814	89.1	-	O = C1NC2 = C(C = CC = C2)C1 = C1NC2 = C(C = CC = C2)C1 = O	9.01	1	2a	Manure: Gipuzkoa 1 & 2, Bizkaia 1 & 2, Araba 1 & 2
4	Propiconazole	Antifungal	C15 H17 Cl2 N3 O2	− 0.52	342.07688	95.3	-	CCCC1COC(CN2C = NC = N2)(O1)C1 = C(Cl)C = C(Cl)C = C1	13.974	1	2a	Soil: Neiker
5	5-Azulenemethanol, 1,2,3,4,5,6,7,8-octahydro-.alpha.,.alpha.,3,8-tetramethyl-, (3S,5R,8S)-	Natural product with antibacterial activity	C15 H26 O	− 0.18	223.2056	-	61.2	CC1CCC(CC2 = C1CCC2C)C(C)(C)O	14.135	1	2b	Manure: Nafarroa_1, Gipuzkoa 1 & 2, Bizkaia 1 & 2, Araba 1 & 2 Soils: Nafarroa 3,4,5 & 6, Araba 1 & 2, Bizkaia 1 & 3
6	Brefeldin A	Antiviral, antibiotic, antifungal, antitumour and herbicidal activity	C16 H24 O4	− 0.83	281.1745		71.8	CC1CCCC = CC2CC(CC2C(C = CC(= O)O1)O)O	12.012	2	2b	Manure: Bizkaia 2, Araba 1
7	8-Hydroxyquinoline	Antibacterial agent, iron chelator, antiseptic drug and antifungal agrochemical	C9 H7 N O	0.26	146.06008	97.1	-	O = C1NC2 = CC = CC = C2C = C1	7.085	1	3	Manure: Gipuzkoa 1 & 2, Bizkaia 1 & 2, Araba 1 & 2
	2-Hydroxyquinoline	Bacterial xenobiotic metabolite	C9 H7 N O	0.28	146.06008	97.8	-	O = C1NC2 = CC = CC = C2C = C1	7.085	1	3	Manure: Gipuzkoa 1 & 2, Bizkaia 1 & 2, Araba 1 & 2
8	Formyl-sulfamethazine	Transformation product of sulfamethazine antibiotic	C13 H14 N4 O3 S	− 0.57	307.08576	77.3	-	CC1 = CC(= NC(= N1)NS(= O)(= O)C2 = CC = C(C = C2)NC = O)C	5.028	1	2a	Manure: Nafarroa_1

^i^Retention time index (RTI) platform (http://rti.chem.uoa.gr/); ^ii^Schymanski scale [44]

## Conclusions

This work shows an improved analytical methodology to determine up to twenty-four antimicrobial and antifungal compounds with marked different physicochemical properties, providing meaningful advance to actually published analytical methodologies and protocols to determine those compounds in soil and manure samples. The methods developed in this work for multiclass antimicrobial analysis in soil and manure samples were successfully validated and proved to be accurate for the trace detection and simultaneous quantification of different antimicrobials in both environmental compartments. In addition, the performed suspect screening allowed the detection of specific antimicrobial TPs as well as other compounds used as antimicrobial agents. The results of this work not only demonstrated the presence of antimicrobials in the environment and the transference of these pharmaceuticals in the manure-soil–plant chain, which puts pressure on bacteria to create resistance, but also showed the presence of their by-products, the effects of which are still unknown.

### Supplementary Information

Below is the link to the electronic supplementary material.

Supplementary file1 (DOCX 429 kb)

Supplementary file2 (XLSX 19 kb)
