# Supplementary material for: Multitarget and suspect screening of antimicrobials in soil and manure by means of QuEChERS — liquid chromatography tandem mass spectrometry
Source: Anal Bioanal Chem. 2023 Aug 23;415(25):6291–310. doi: 10.1007/s00216-023-04905-2 (PMC10558387; doi:10.1007/s00216-023-04905-2)
Supplement: Supplementary file 1 — Supplementary file1 (DOCX 429 kb) [file 216_2023_4905_MOESM1_ESM.docx]

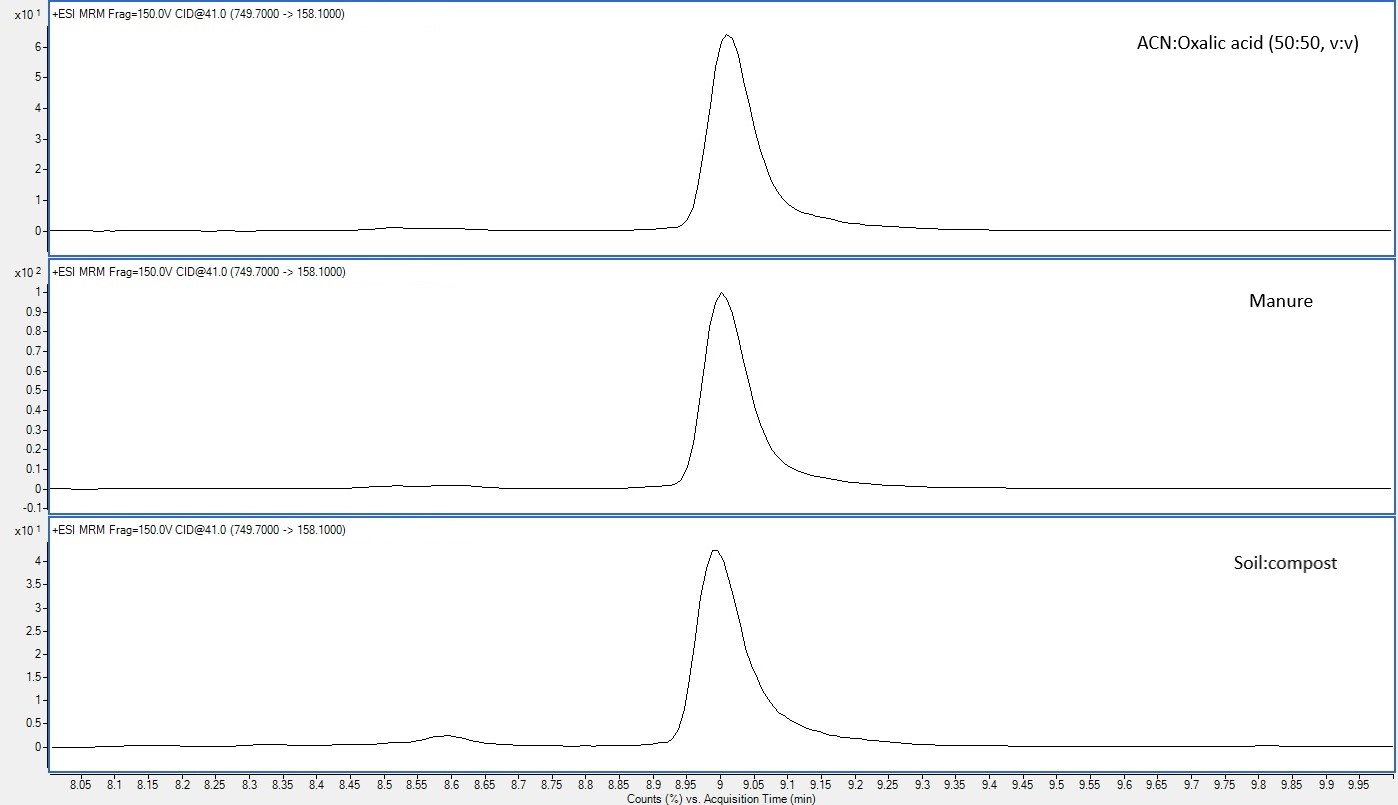


**Figure S1**. Dynamic multiple reaction monitoring (DMRM) chromatograms for azithromycin, as representative of the macrolides, at 25 µg·kg^-1^ in a reference standard solution in ACN:oxalic acid (50:50, v:v), in manure and soil:compost samples.


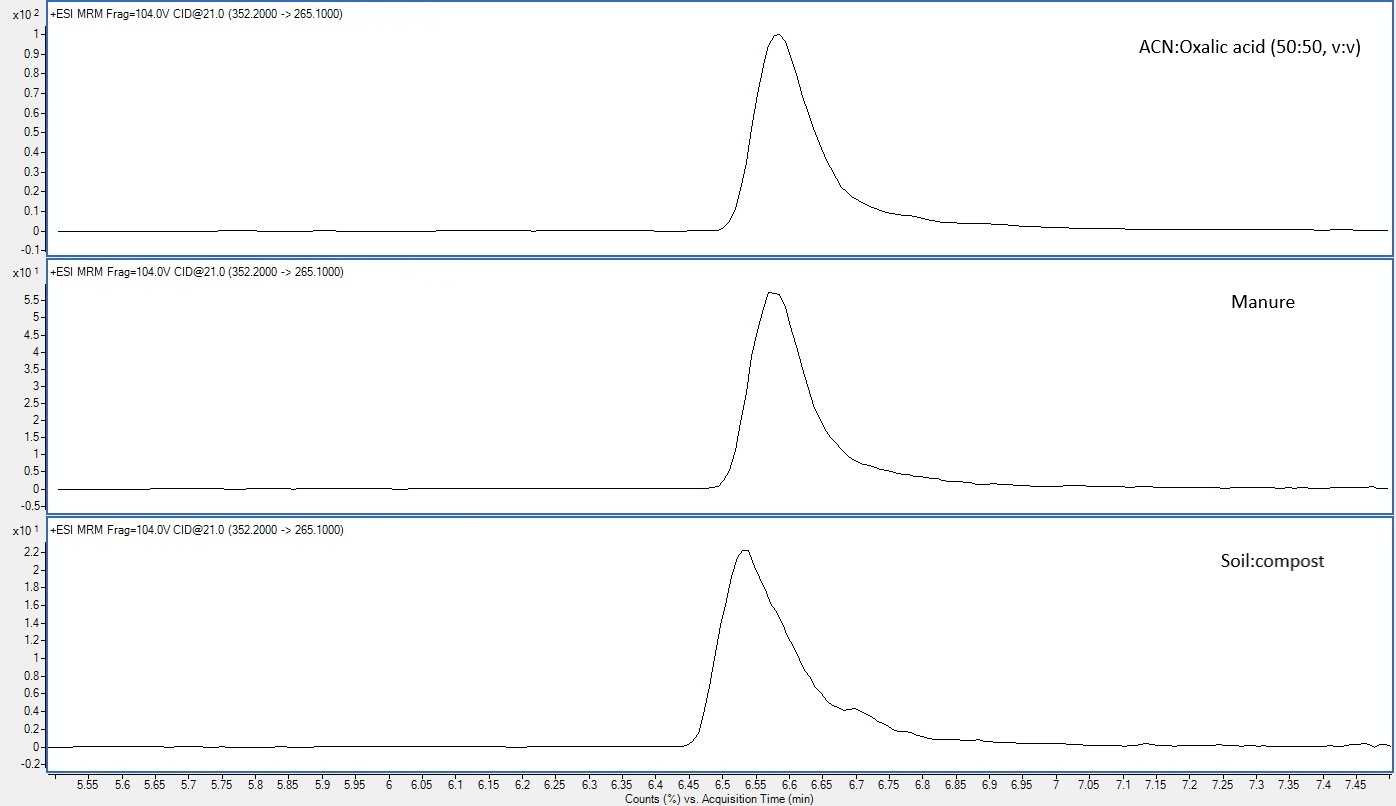


**Figure S2**. Dynamic multiple reaction monitoring (DMRM) chromatograms for lomefloxacin, as representative of the (fluoro)quinolones, at 25 µg·kg^-1^ in a reference standard solution in ACN:oxalic acid (50:50, v:v), in manure and soil:compost samples.


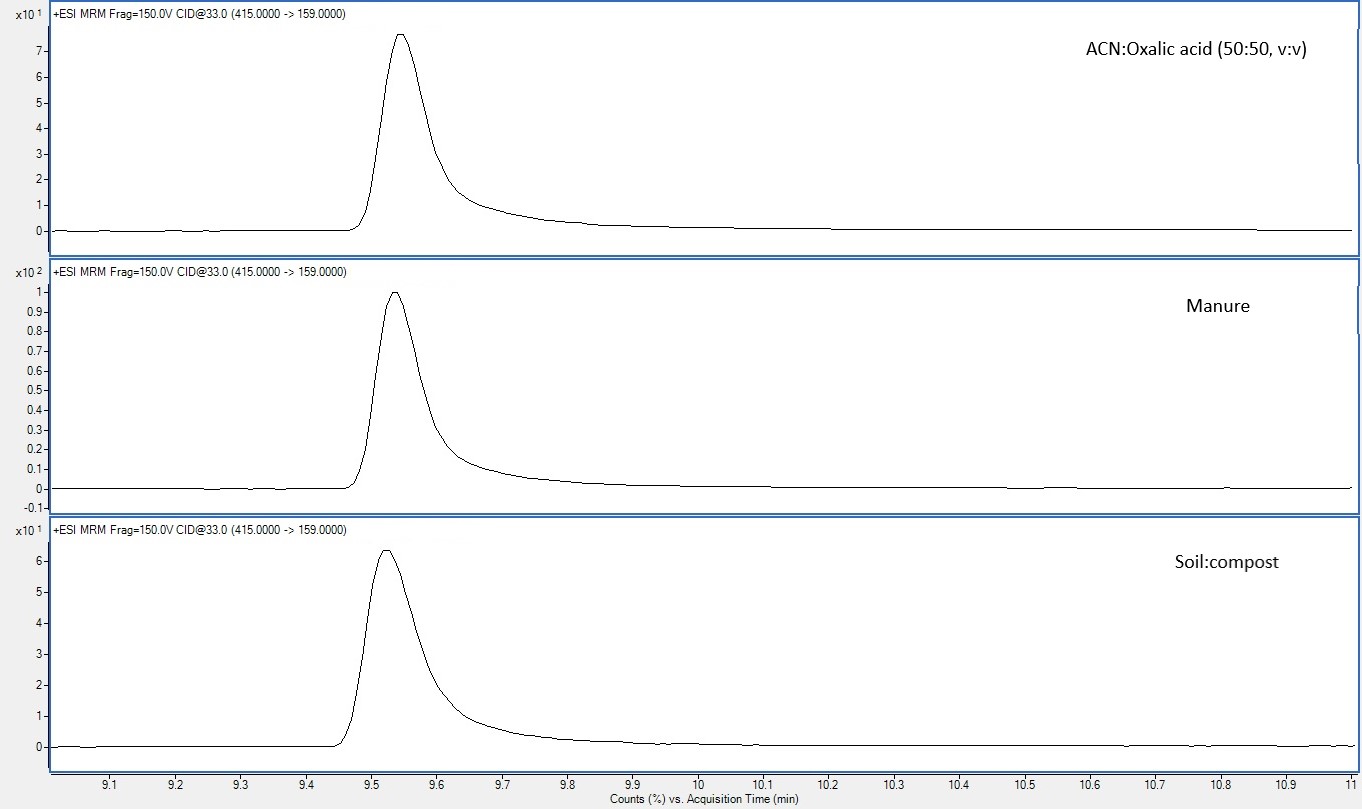


**Figure S3**. Dynamic multiple reaction monitoring (DMRM) chromatograms for miconazole at 25 µg·kg^-1^ in a reference standard in ACN:oxalic acid (50:50, v:v), in manure and soil:compost samples.


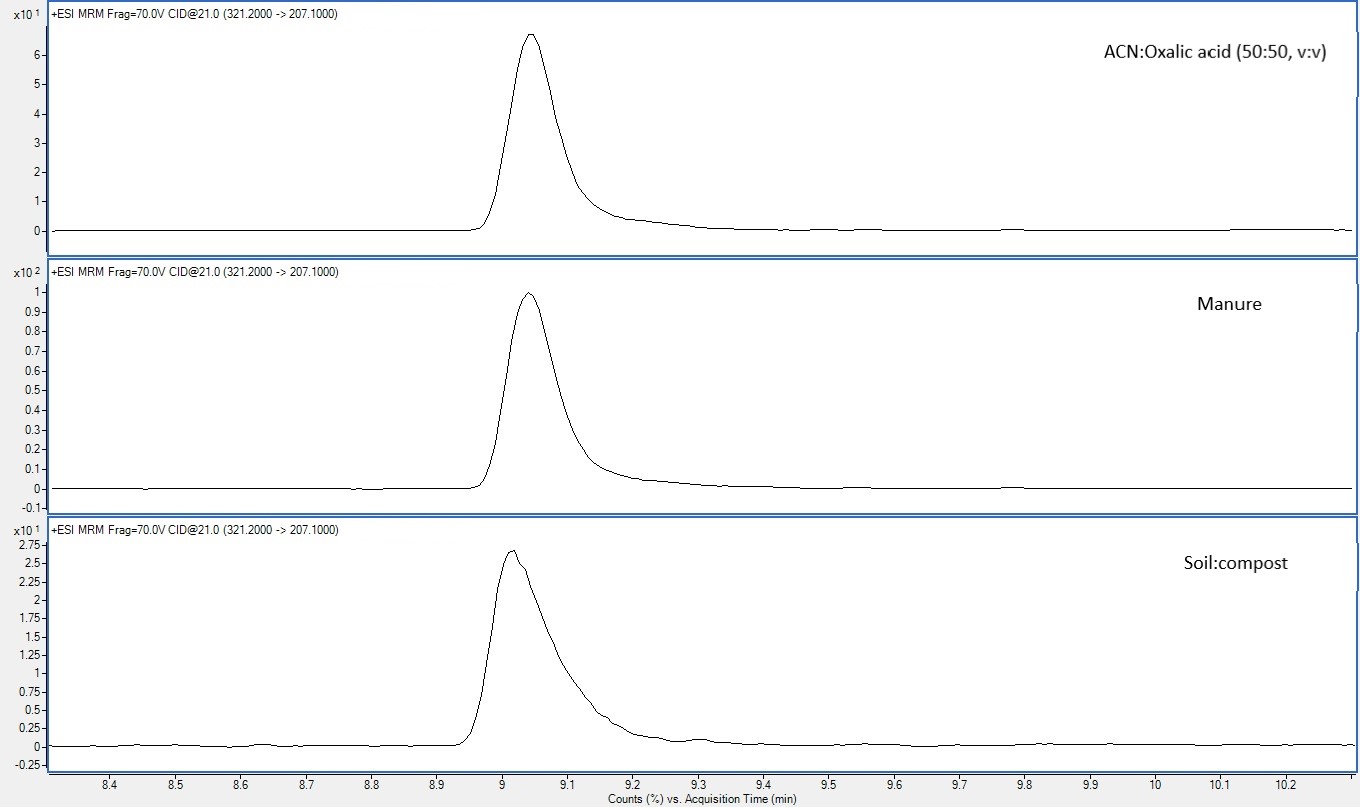


**Figure S4**. Dynamic multiple reaction monitoring (DMRM) chromatograms for mycophenolic acid at 25 µg·kg^-1^ in a reference standard solution in ACN:oxalic acid (50:50, v:v), in manure and soil:compost samples.

**Table S1**. Distributor and specific physicochemical properties for the target antibiotics, antifungals and surrogate standards.

| **Antibiotics** | **Molecular formula** | **Purity (%)** | **Distributor** | **Solvent** | **pKa** [1] **(sa/sb)** |
| --- | --- | --- | --- | --- | --- |
| Sulfadiazine | C_10_H_10_N_4_O_2_S | 99 | Sigma-Aldrich^a^ | ACN | 7.0; 2.0 |
| Sulfamethazine | C_12_H_14_N_4_O_2_S | 100 | Sigma-Aldrich^a^ | ACN | 7.0; 2.0 |
| Sulfamethoxazole* | C_10_H_11_N_3_O_3_S | 98 | Sigma-Aldrich^a^ | ACN | 6.2; 2.0 |
| Sulfapyridine | C_11_H_11_N_3_O_2_S | 100 | Sigma-Aldrich^a^ | ACN | 6.2; 2.1 |
| Sulfathiazole | C_9_H_9_N_3_O_2_S_2_ | 100 | Sigma-Aldrich^a^ | ACN | 6.9; 2.0 |
| Thiabendazole | C_10_H_7_N_3_S | 100 | Sigma-Aldrich^a^ | MeOH | 10.3; 4.1 |
| Trimethoprim | C_14_H_18_N_4_O_3_ | 98 | Sigma-Aldrich^a^ | MeOH | 17.33; 7.16 |
| Danofloxacin | C_19_H_20_FN_3_O_3_ | 100 | Sigma-Aldrich^a^ | MeOH+NaOH | 5.70; 6.7 |
| Enrofloxacin | C_19_H_22_FN_3_O_3_ | 98 | Sigma-Aldrich^a^ | MeOH+NaOH | 5.6; 7.2 |
| Fluconazole | C_13_H_12_F_2_N_6_O | 100 | Sigma-Aldrich^a^ | ACN | 12.7; 2.3 |
| Flumequine | C_14_H_12_FNO_3_ | 99 | Sigma-Aldrich^a^ | MeOH+NaOH | 5.8; -4.3 |
| Lomefloxacin | C_17_H_19_F_2_N_3_O_3_ | >99 | Sigma-Aldrich^a^ | MeOH+NaOH | 5.50; 8.80 |
| Mycophenolic acid* | C_17_H_20_O_6_ | 99 | Sigma-Aldrich^a^ | MeOH | 3.57; -4.1 |
| Ofloxacin | C_18_H_20_FN_3_O_4_ | 100 | Sigma-Aldrich^a^ | MeOH+NaOH | 5.4; 6.7 |
| Pefloxacin | C_17_H_20_FN_3_O_3_ | >99 | Sigma-Aldrich^a^ | MeOH+NaOH | 5.6; 7.0 |
| Azithromycin | C_38_H_72_N_2_O_12_ | 95 | Sigma-Aldrich^a^ | ACN | 12.4; 9.6 |
| Clarithromycin | C_38_H_69_NO_13_ | 100 | Sigma-Aldrich^a^ | DMSO | 12.5; 9.0 |
| Erythromycin | C_37_H_67_NO_13_ | 100 | Sigma-Aldrich^a^ | ACN | 12.5; 9.0 |
| Roxithromycin | C_41_H_76_N2O_15_ | 100 | Sigma-Aldrich^a^ | ACN | 12.5; 9.1 |
| Tetracycline | C_22_H_24_N_2_O_8_ | 88 | Sigma-Aldrich^a^ | ACN | 3.3; 9.3 |
| Oxytetracycline | C_22_H_24_N_2_O_9_ | 98 | Sigma-Aldrich^a^ | MeOH | 3.2; 8.2 |
| Doxycycline | C_22_H_24_N_2_O_8_ | 97 | Sigma-Aldrich^a^ | MeOH | 3.3; 8.3 |
| Chlortetracycline | C_22_H_23_ClN_2_O_8_ | 93 | Sigma-Aldrich^a^ | MeOH | 3.0; 9.0 |
| Miconazole | C_18_H_14_Cl_4_N_2_O | 100 | Sigma-Aldrich^a^ | MeOH | nd; 6.5 |
| [^2^H_6_]-Tetracycline | C_22_H_18_D_6_N_2_O_8_ | >80 | trc Canada^b^ | ACN | - |
| [^13^C_6_]-sulfamethoxazole | C_10_H_11_N_3_O_3_S | 98 | LGC Standards^c^ | MeOH | - |
| [^2^H_4_]-Sulfamethazine | C_12_H_10_D_4_N_4_O_2_S | 99 | A2S Standards^d^ | ACN | - |
| [^2^H_5_]-Enrofloxacin | C_19_H_17_D_5_FN_3_O_3_ | >99 | Sigma-Aldrich^a^ | MeOH | - |
| [^2^H_7_]-Roxithromycin | C_41_H_69_D_7_N_2_O_15_ | 99 | trc Canada^b^ | MeOH | - |

^a^ St. Louis, Missouri, USA

^b^ Toronto, Ontario, Canada

^c^ Augsburg, Bavaria, Germany

^d^ Saint-Jean-d'Illac, Aquitanie, France

**Table S2.** Fragmentor voltages (V), collision energies (eV) and parent and product ions *(m/z)* for the target analytes and surrogates.

| **Antimicrobials** | **Parent ion (m/z)** | **Product ion (m/z)** | **Frag. Voltage (V)** | | **Collision energy (eV)** | |
| --- | --- | --- | --- | --- | --- | --- |
| Sulfadiazine | 251.1 | 156.0/92.1 | 86 | 13/29 | |  |
| Sulfamethazine | 279.2 | 186.0/124.0 | 86 | 13/25 | |  |
| Sulfamethoxazole | 254.1 | 156.0/108.1 | 86 | 13/25 | |  |
| Sulfapyridine | 250.1 | 156.0/108.1 | 86 | 13/25 | |  |
| Sulfathiazole | 256.1 | 156.0/108.1 | 70 | 9/21 | |  |
| Thiabendazole | 202.2 | 175.0/131.1 | 118 | 25/37 | |  |
| Trimethoprim | 291.2 | 230.1/123.1/261.0 | 118 | 21/25/25 | |  |
| Danofloxacin | 358.2 | 340.2/82.1 | 104 | 21/45 | |  |
| Enrofloxacin | 360.3 | 342.1/286.1 | 102 | 21/37 | |  |
| Fluconazole | 307.2 | 220.1 /238.1 | 102 | 17/13 | |  |
| Flumequine | 262.1 | 244.1/202.0 | 86 | 17/33 | |  |
| Lomefloxacin | 352.2 | 265.1/308.2 | 104 | 21/13 | |  |
| Mycophenolic acid | 321.2 | 207.1/303.1 | 70 | 21/5 | |  |
| Ofloxacin | 362.3 | 318.1/261.1 | 118 | 17/29 | |  |
| Pefloxacin | 334.2 | 316.1/290.1 | 104 | 17/13 | |  |
| Azithromycin | 749.7 | 158.1/116.1 | 150 | 41/45 | |  |
| Clarithromycin | 748.8 | 158.1/590.4 | 150 | 29/13 | |  |
| Erythromycin | 734.8 | 158.1/576.3 | 150 | 45/45 | |  |
| Roxithromycin | 837.8 | 679.4/158.1 | 150 | 17/33 | |  |
| Tetracycline | 445.2 | 410.1/427.1 | 135 | 17/9 | |  |
| Oxytetracycline | 461.2 | 426.1/443.1 | 120 | 17/9 | |  |
| Doxycycline | 445.2 | 428.1/154.1 | 135 | 17/33 | |  |
| Chlortetracycline | 479.1 | 462.1/444.1 | 135 | 17/21 | |  |
| Miconazole | 415.0 | 159.0/227.0 | 150 | 33/17 | |  |
| **Surrogates** |  |  |  |  | |  |
| [^2^H_5_]-enrofloxacin | 365.0 | 347.0/321.0 | 104 | 37/37 | |  |
| [^2^H_4_]-sulfamethazine | 283.1 | 185.9/124.0 | 102 | 24/33 | |  |
| [^13^C_6_]-sulfamethoxazole | 260.1 | 162.0/114.1 | 105 | 13/25 | |  |
| [^2^H_6_]-tetracycline | 451.2 | 433.2/416.2 | 120 | 9/17 | |  |
| [^2^H_7_]-roxithromycin | 844.6 | 158.1/686.5 | 150 | 37/21 | |  |

**Table S3**. Figures of merit of UHPLC-MS/MS measurements: linear range, determination coefficients (r^2^), repeatability and intermediate repeatability expressed as relative standard deviation (RSD %) for 2.5 μg·kg^-1^ concentration level, and instrumental limits of quantification (LOQs).

| **Antimicrobials** | **LOQ_INS_ (µg·kg^-1^)** | **r^2^** | **Linear range (μg·kg^-1^)** | **Repeatability 2.5 μg·kg^-1^ (RSD%)** | **Intermediate repeatability  2.5 μg·kg^-1^ (RSD%)** |
| --- | --- | --- | --- | --- | --- |
| Sulfadiazine | 0.22 | 0.9996 | LOQ_INS_-84.01 | 2 | 2 |
| Sulfamethazine | 0.22 | 0.9991 | LOQ_INS_-84.93 | 9 | 7 |
| Sulfamethoxazole | 0.20 | 0.9987 | LOQ_INS_-84.55 | 6 | 10 |
| Sulfapyridine | 0.22 | 0.9998 | LOQ_INS_-84.51 | 3 | 2 |
| Sulfathiazole | 0.20 | 0.9995 | LOQ_INS_-84.33 | 1 | 2 |
| Thiabendazole | 0.21 | 0.9986 | LOQ_INS_-79.70 | 1 | 1 |
| Trimethoprim | 0.21 | 0.9999 | LOQ_INS_-77.13 | 4 | 3 |
| Danofloxacin | 0.45 | 0.9986 | LOQ_INS_-80.32 | 2 | 4 |
| Enrofloxacin | 0.22 | 0.9997 | LOQ_INS_-81.36 | 5 | 4 |
| Fluconazole | 0.45 | 0.9990 | LOQ_INS_-83.17 | 4 | 4 |
| Flumequine | 0.21 | 0.9999 | LOQ_INS_-83.07 | 1 | 4 |
| Lomefloxacin | 0.22 | 0.9993 | LOQ_INS_-82.30 | 3 | 5 |
| Mycophenolic acid | 0.18 | 0.9994 | LOQ_INS_-78.71 | 9 | 6 |
| Ofloxacin | 0.92 | 0.9997 | LOQ_INS_-84.35 | 5 | 4 |
| Pefloxacin | 0.19 | 0.9996 | LOQ_INS_-82.72 | 8 | 8 |
| Azithromycin | 0.22 | 0.9998 | LOQ_INS_-83.77 | 1 | 2 |
| Clarithromycin | 0.32 | 0.9999 | LOQ_INS_-121.23 | 3 | 2 |
| Erythromycin | 0.91 | 0.9980 | LOQ_INS_-83.55 | 5 | 7 |
| Roxithromycin | 0.91 | 0.9993 | LOQ_INS_-83.17 | 2 | 3 |
| Tetracycline | 0.22 | 0.9998 | LOQ_INS_-83.88 | 4 | 5 |
| Oxytetracycline | 0.21 | 0.9987 | LOQ_INS_-81.89 | 5 | 8 |
| Doxycycline | 0.21 | 0.9995 | LOQ_INS_-80.22 | 4 | 4 |
| Chlortetracycline | 0.44 | 0.9972 | LOQ_INS_-80.49 | 5 | 8 |
| Miconazole | 0.84 | 0.9997 | LOQ_INS_-80.52 | 1 | 1 |

**Table S4**. Antimicrobial classes, sample treatment steps and conditions, linearity, procedural limits of quantification (LOQs), apparent recoveries and precision expressed as relative standard deviation (RSD %) of analytical methods developed for multiresidue/multiclass antimicrobials analysis in soil and manure samples.

## Pre-treatment and extraction of plant samples

Fresh, crushed and homogenised plant samples (10 g) were weighed in a 50 mL polypropylene centrifuge tube and spiked with 200 μL of a 5 mg·kg^-1^ stock solution containing the surrogate compounds. Afterwards, samples were vortexed (2000 cycles⋅min^-1^, 10 min) and kept in the darkness for 30 min at room temperature. ACN (10 mL), a ceramic homogeniser and the extraction salts (4 g anhydrous Na_2_SO_4_, 1 g NaCl, 0.5 g anhydrous H_3_Cit and 0.049 g anhydrous Na_2_HPO_4_) were added. The mixture was then shaken manually and degasified by opening the centrifuge tube, until no gas was released. All samples were vortexed (2000 cycles⋅min^-1^, 8 min) and centrifuged (4000 rpm, 5 min) at 10–15◦C. A representative aliquot of 1 mL of the extractant was transferred to a 50 mL centrifuge tube containing 10 mg PSA, 25 mg C_18_ and 150 mg de Na_2_SO_4_, and the mixture was then vortexed (2000 cycles⋅min^-1^, 1 min) and centrifuged (4000 cycles⋅⋅min^-1^, 5 min) at 10–15 ◦C. Aliquots of 500 μL were reconstituted in 1 mL of a 1:1 (v/v) ACN:oxalic acid (aq., 0.01 mol·L^-1^, pH 2) and filtered through 0.22 μm polypropylene filters (Clarify-PP, Phenomenex, Torrance, California, USA) before UHPLC- MS/MS analysis [2].

1. Browsing Drugs | DrugBank Online. https://go.drugbank.com/drugs. Accessed 19 Oct 2022

2. Vergara-Luis I, Báez-Millán JC, Baciero I, et al (2022) Comparison of conventional and dispersive solid phase extraction clean-up approaches for the simultaneous analysis of tetracyclines and sulfonamides in a variety of fresh vegetables. Talanta 124192. https://doi.org/10.1016/j.talanta.2022.124192
